# Fucoidan P Alleviates Sarcopenic Obesity by Regulating Muscle Protein and Energy Metabolism

**DOI:** 10.7150/ijbs.125462

**Published:** 2026-04-08

**Authors:** Jong-Yeon Kim, Sung-Min Kim, Sanghoon Lee, Ju-Hong Jeon, Eun-Jung Park, Hae-Jeung Lee

**Affiliations:** 1 Department of Food Science and Biotechnology, Gachon University, Gyeonggi-do 13120, Republic of Korea.; 2 Department of Physiology & Biomedical Sciences and Institute of Human-Environment Interface Biology, Seoul National University College of Medicine, Seoul 03080, Republic of Korea.; 3 Department of Food and Nutrition, Gachon University, Gyeonggi-do 13120, Republic of Korea.; 4 Institute for Aging and Clinical Nutrition research, Gachon University, Gyeonggi-do 13120, Republic of Korea.; 5 Department of Health Sciences and Technology, GAIHST, Gachon University, Incheon 21999, Republic of Korea.; 6 Gachon Biomedical Convergence Institute, Gachon University Gil Medical Center, Incheon 21565, Republic of Korea.

**Keywords:** Fucoidan, sarcopenic obesity, muscle atrophy, energy metabolism, inflammatory response

## Abstract

Sarcopenic obesity (SO) is a complex condition involving increased fat accumulation along with reduced muscle mass and impaired muscle function; however, there are currently no approved pharmacological interventions targeting these pathological features. Akt and AMPK are key signaling pathways regulating muscle homeostasis and energy metabolism. Natural compounds that modulate these pathways may offer a promising multi-targeted therapeutic strategy. This study investigated the effects of Fucoidan P on SO and its underlying molecular pathways, while assessing translational relevance using clinical datasets. Fucoidan P decreased body weight and fat mass and size while improving grip strength, muscle mass, and fiber size during high-fat diet feeding. Fucoidan P mitigated muscle atrophy through phosphorylation of Akt, mTOR, and FOXO3a, and enhanced energy metabolism by activating the AMPK/SIRT1/PGC-1α pathway. Moreover, Fucoidan P downregulated PDE5A and PTGS1, which are identified differentially expressed genes-related to inflammation of clinical datasets, and inhibited pro-inflammatory cytokines by suppressing NF-κB pathway. Taken together, these findings demonstrate that Fucoidan P alleviates SO by coordinately regulating muscle protein homeostasis, energy metabolism, and inflammatory responses through a network of interconnected Akt and AMPK/PGC-1α, and NF-κB signaling pathways, suggesting its potential as a multi-target therapeutic agent for SO.

## Introduction

Sarcopenic obesity (SO) refers to a complex syndrome in which fat accumulation increases simultaneously with a decline in muscle mass and function 1. This condition arises from the interaction between sarcopenia and obesity, resulting in synergistic effects that adversely affect skeletal muscle 2. Research indicates that the prevalence of SO is nearly double that of general obesity 3, and SO is linked to a 24% increased risk of all-cause mortality, when contrasted with having either obesity or sarcopenia alone 4. However, appropriate diagnostic criteria and specific therapeutic strategies have yet to be established 5. Therefore, SO is increasingly recognized as a significant health concern.

Skeletal muscle accounts for the majority of total energy expenditure, and its preservation is vital for mitigating metabolic diseases, including SO 6. Muscle metabolism is regulated by various proteins and signaling pathways, with the Akt and AMP-activated protein kinase (AMPK)/peroxisome proliferator-activated receptor gamma coactivator alpha (PGC-1α) pathways serving as representative mechanisms 7, 8.

Muscle homeostasis is tightly regulated through the Akt signaling pathway, which stimulates protein synthesis by activating mammalian target of rapamycin (mTOR) while suppressing muscle degradation through the inhibition of forkhead box O (FOXO)-mediated ubiquitin-proteasome activity 8. AMPK senses the energy status within cells and regulates multiple biological processes within skeletal muscle, including muscle atrophy, lipid metabolism, secretion of myokines, and the regulation of mitochondrial function 9. PGC-1α, which functions downstream of AMPK as a transcriptional coactivator, influences the activity of various genes that participate in energy metabolism, including fatty acid oxidation and fat browning, by interacting with transcription factors 10.

Fucoidan, a polysaccharide composed of sulfates, uronic acid, and monosaccharides (galactose, fucose, mannose) 11, exhibits diverse biological activities, such as anticoagulant, antiviral, and immunomodulatory effects 12. We previously demonstrated the anti-muscle atrophy and anti-inflammatory activities of high-molecular-weight fucoidan (Fucoidan P) in palmitate (PA)-treated C2C12 myotubes 13; however, the underlying molecular pathways involved in the SO-ameliorating effects of Fucoidan P remain to be elucidated. Therefore, we investigated whether Fucoidan P improved high-fat diet (HFD)- and PA-induced SO via multifaceted mechanisms. We further analyzed datasets from the Gene Expression Omnibus (GEO) database and validated our findings in both animal and cellular models, thereby assessing the translational relevance of our results to clinical conditions.

## Materials and Methods

### Materials

C2C12 myoblasts were purchased from the American Type Culture Collection (Manassasm, VA, USA). Dulbecco's Modified Eagle's Medium (DMEM) was purchased from Corning Inc. (Corning, NY, USA). Fetal bovine serum (FBS) and antibiotic-antimycotic solution were purchased from Gibco BRL (Grand Island, NY, USA). Horse serum (HS), sodium palmitate (PA), Akti-1/2, compound C, Oil Red O solution, 2-propanol, protease inhibitor, and quercetin were purchased from Sigma-Aldrich (St. Louis, MO, USA). 4% paraformaldehyde and RIPA buffer were obtained from iNtRON Biotechnology (Seongnam, Korea). EX-527 was purchased from Selleckchem (Houston, TX, USA) and phosphatase inhibitors were purchased from Thermo Fisher Scientific (Waltham, MA, USA).

### Preparation of high-molecular-weight fucoidan (Fucoidan P)

Fucoidan P was obtained from Haerim Fucoidan Co. (Wando, Korea). Fucoidan P was extracted from the sporophyll of *Undaria pinnatifida* using standard production processes 14. Briefly, after inspecting the raw material of *Undaria pinnatifida* sporophyll, it was extracted with water. The extract was purified through alginate coagulation, filter press filtration, and ultrafiltration. Subsequently, Fucoidan P was fractionated using high-molecular-weight filtration systems. Fucoidan P powder was obtained through concentration, sterilization, freeze-drying, and pulverization. The sugar composition of Fucoidan P is as follows: 22 ± 5% fucose, 25 ± 5% galactose, and 2 ± 2% mannose, with a sulfate content of 30 ± 3%. It has a purity exceeding 90% and an average molecular weight of 232,404 Da.

### Animal experiment

Male C57BL/6 mice (5 weeks old) were purchased from Orient Bio Inc. (Seongnam, Korea) and were maintained at 20-25°C with 50-55% humidity under a 12-h light-dark cycle. The animal experiment was conducted in accordance with the National Research Council's Guide for the Care and Use of Laboratory Animals. All animal procedures were approved by the Institutional Animal Care and Use Committee of Gachon University (GU1-2023-IA0004).

After a one-week adaptation period, the mice were fed either a normal-fat diet (NFD; Research Diets Inc., New Brunswick, NJ, USA) or a 45% HFD (Research Diets Inc.) for eight weeks to induce SO (Fig. S1). Water was provided ad libitum. Following SO induction, the mice were randomly assigned to six groups. The control groups included NC (NFD control), HC (HFD control), and PC (positive control). The treatment groups included FL, FM, and FH groups, which were administered Fucoidan P at low, medium, and high doses, respectively. All treatments were administered orally once per day over a six-week period. The NC and HC groups received saline, whereas the PC group was given quercetin at 50 mg/kg b.w. The FL, FM, and FH groups were given Fucoidan P at doses of 50, 100, and 200 mg/kg b.w., respectively. The NC group was fed an NFD, while all other groups received an HFD. Body weight was recorded once a week, and food intake was measured twice a week.

### Sample collection

At study end, mice were anesthetized with CO_2_ and sacrificed after whole blood collection. The skeletal muscles, including the quadriceps femoris (Quad), gastrocnemius (Gas), extensor digitorum longus (EDL), soleus (Sol), and tibialis anterior (TA), as well as white fat, including epididymal (EpF), retroperitoneal (ReF), and subcutaneous (SuF) fat was separated and weighed. Whole blood was centrifuged at 12,000 rpm, and the serum was collected and stored at -80°C. All tissues were stored at -80°C.

### Biochemical analysis

Triglyceride (TG), total cholesterol (TC), high-density lipoprotein cholesterol (HDL-C), aspartate transaminase (AST), alanine aminotransferase (ALT), lactate dehydrogenase (LDH), and creatine phosphokinase (CPK) levels were measured using an automatic analyzer (Beckman Coulter, USA). Low-density lipoprotein cholesterol (LDL-C) was calculated as TC - (TG/5 + HDL-C).

### Micro-computed tomography (micro-CT)

Micro-CT was performed *in vivo*. After anesthesia by intraperitoneal injection of a mixture of alfaxalone and xylazine, the abdomen and hindlimbs were scanned using micro-CT (Quantum GX2, PerkinElmer, Inc., Switzerland). Regions of interest were set for abdominal fat and hindlimbs to measure fat and muscle lean mass. Visceral fat (VF) and SuF were shown in red and yellow colors, respectively. Orange and cyan colors were used to indicate left and right hindlimbs, respectively.

### Hematoxylin and eosin (H&E) staining

The EpF, Quad, and Gas were fixed with formaldehyde, embedded in paraffin, and sectioned at a thickness of 4 µm. The paraffin was removed using xylene and ethanol. Tissue samples were stained with H&E. Stained nuclei and cytoplasm were observed under an optical microscope after dehydration and clearing with ethanol and xylene. The cross-sectional area (CSA) of fat and muscle fibers were quantified using ImageJ software (NIH, USA).

### Grip strength

The grip strength of the mice was measured thrice using a grip strength meter (47200; Ugo Basile, Italy) after six weeks of administration. Briefly, we held the mice by their tails, ensuring that all limbs were positioned on the grid of a grip strength meter. Subsequently, the grip strength was measured during tail pull. Grip strength was expressed as absolute grip strength normalized to body weight.

### Enzyme-linked immunosorbent assay (ELISA)

Serum tumor necrosis factor-alpha (TNF-α), interleukin-6 (IL-6), and IL-1β were analyzed using ELISA kits (R&D systems, Minneapolis, MN, USA), following the manufacturer's directions.

### Cell culture and treatment

C2C12 myoblasts were cultured in DMEM containing 10% FBS and 1% antibiotic-antimycotic solution. To induce myogenic differentiation, when the cells reached 100% confluence, DMEM containing 10% FBS was replaced with DMEM containing 2% HS. Over a six-day period, 2% HS-DMEM was refreshed every other day. C2C12 myotubes were treated with 0.75 mM PA and different concentrations of Fucoidan P.

Three inhibitors were used to assess the Akt and AMPK/sirtuin1 (SIRT1) signaling pathways; Akti-1/2 for Akt, EX-527 for SIRT1, and Compound C for AMPK. Based on the dose-dependent results, 200 μg/mL was selected as the representative Fucoidan P concentration for inhibitor experiments. We treated C2C12 myotubes with 0.75 mM PA and 200 μg/mL Fucoidan P in the absence or presence each inhibitor.

### Oil Red O staining

C2C12 myotubes were treated with Fucoidan P and PA for 24 h. Myotubes were fixed in 4% paraformaldehyde and stained with Oil Red O solution in the dark. Images of stained myotubes were captured using a microscope (Nikon, Japan). Oil Red O staining dye was dissolved in 2-propanol, and lipid accumulation was quantified by measuring the absorbance at 500 nm.

### Reactive oxygen species (ROS) assay

C2C12 myotubes were treated with Fucoidan P and PA for 24 h. To avoid background fluorescence, 2% HS-DMEM without phenol red was used. Myotubes were stained with 2',7'dichlorofluorescin diacetate solution (Abcam, Cambridge, UK) in the dark. Images of stained myotubes were captured using a fluorescence microscope (Korea Lab Tech, Korea). Fluorescence intensity was quantified using ImageJ software (NIH).

### Luciferase assay

C2C12 myoblasts were plated onto a 96-well white plate and co-transfected with 3×κB-Luc 15 or 3×AP1pGL3 (a gift from Alexander Dent, Addgene plasmid #40,342; Addgene, USA) and pNL1.1. TK vector (Promega, Madison, Wisconsin, USA), served as an internal control, using FuGENE® HD Transfection Reagent (Promega). Following transfection for 24 h, the cells were co-treated with Fucoidan P and PA for an additional 24 h. Luciferase activity was assessed using a commercial kit (Promega).

### RNA extraction and qRT-PCR analysis

SuF, Quad, and Gas tissues were homogenized using a homogenizer (Polyton PT-MR 3100, Switzerland). Total RNA from tissues and cells were extracted using Easy-spin™ total RNA extraction kit (iNtRON Biotechnology). Extracted RNA was quantified at a concentration of 50 ng/μL using Take3 Micro-Volume plate (BioTek Instruments, USA). Complementary DNA was synthesized and amplified using primers (Table S1) and TB Green Premix Ex Taq II (TaKaRa Bio, Japan). mRNA expression levels were analyzed using a QuantStudio 3 real-time PCR instrument (Thermo Fisher Scientific) and normalized to GAPDH.

### Protein extraction and western blot analysis

C2C12 myotubes and homogenized Quad tissues were lysed in RIPA buffer supplemented with protease and phosphatase inhibitors. The protein concentration was quantified using a BCA protein assay kit (TaKaRa Bio). Proteins were loaded in SDS-PAGE gels and transferred onto PVDF membranes. Membranes were then incubated with primary antibodies (Table S2). After washing, the membranes were incubated with secondary antibodies (Promega). Protein bands were visualized using an enhanced chemiluminescence reagent (iNtRON Biotechnology) and imaged with an ImageQuant™ LAS 500 system (GE Healthcare, Sweden). Protein expression levels of non-phosphorylated proteins were normalized to GAPDH, and phosphorylated proteins were quantified as the ratio of phosphorylated form to the corresponding total protein level. Data are presented as fold changes relative to NC or vehicle control.

### Data acquisition

RNA-seq datasets (GSE63887 and GSE81965) were downloaded from the NCBI GEO (https://www.ncbi.nlm.nih.gov/geo/). The GSE63887 dataset included three participants without obesity and with normal glucose tolerance (3 males, age 51.7 ± 2.4 years, BMI 24.0 ± 0.3 kg/m^2^), while the GSE81965 dataset included three participants with obesity and normal glucose tolerance (3 males, age 48.0 ± 6.2 years, BMI 36.1 ± 2.8 kg/m^2^). Isolated skeletal muscle precursor cells obtained from human muscle tissue through biopsy were differentiated in vitro and subsequently used for RNA-seq analysis. Differentially expressed gene (DEG) and fold changes between groups were analyzed using DESeq2 (v.1.44.0) 16. Genes with an adjusted p-value < 0.05 were considered significant DEGs. Principal component analysis (PCA) and hierarchical clustering analysis were performed to identify differences in gene expression between participants without obesity and those with obesity. We performed PCA after applying variance-stabilizing transformation (VST) to the data normalized with DESeq2. Subsequently, we identified 5,000 DEGs sorted by adjusted p-value in DESeq2 and conducted hierarchical clustering analysis using the R package ComplexHeatmap (v.2.20.0) 17. Volcano plots illustrated the log2 fold change and adjusted p-value of DEGs, highlighting the significant genes.

To investigate the signaling pathways that are specific to the participants with obesity, we conducted a Weighted Concept Signature Enrichment Analysis (WCSEA) using the IndepthPathway R package (v.1.0) 18. The gene signature data were derived from the hallmark gene sets obtained from the Molecular Signatures Database (MSigDB) 19. We then calculated the average normalized enrichment score (NES) and false discovery rate (FDR) from pairwise WCSEA analyses, applying FDR q-value of less than 0.05 to identify significantly enriched pathways. For data visualization, we employed the ggplot2 package (v.3.5.1) 20.

### Statistical analysis

All results are presented as the mean ± standard deviation of at least three independent experiments. Differences were analyzed using one-way analysis of variance (ANOVA) followed by Tukey's HSD test. Statistical significance was set at *p* < 0.05. All statistical analyses were conducted using the Statistical Package for the Social Sciences (SPSS) 28.0 (SPSS Inc., USA). All graphs were visualized using GraphPad Prism 10 software (GraphPad Software Inc., USA).

## Results

### Fucoidan P suppresses obesity in HFD-fed mice

**Figure 1:**
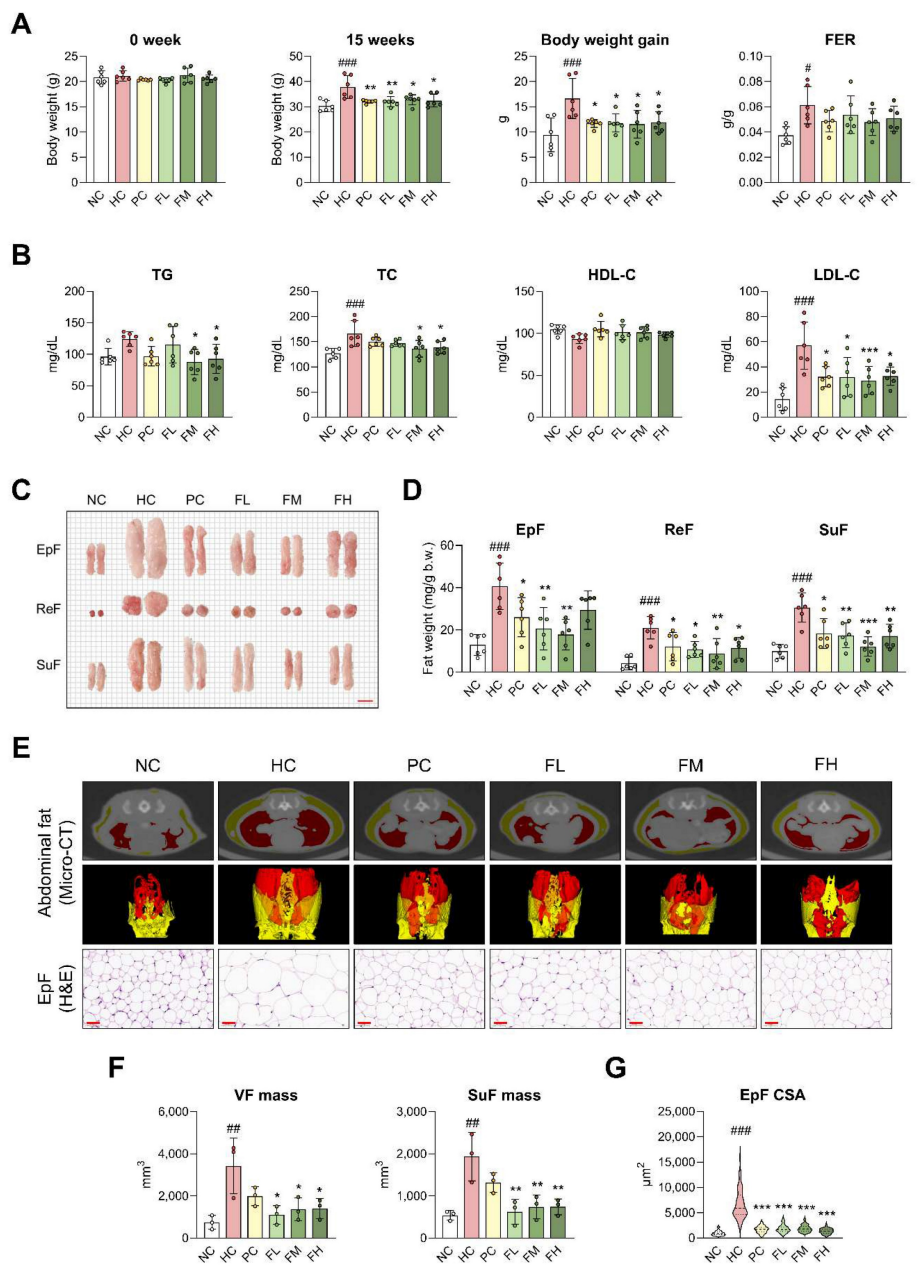
** Effects of Fucoidan P on obesity in HFD-fed mice.** The mice were fed an HFD for 8 weeks, and then orally administered Fucoidan P or quercetin or saline for 6 weeks. (A) Body weight at 0 week and 15 weeks, body weight gain from 0 to 15 weeks, and FER. (B) Serum lipid profiles. (C) Representative images of white fat (scale bar = 1 cm). (D) White fat weight (mg/g b.w.). (E) Representative micro-CT images of abdominal fat and representative H&E-stained images of EpF (scale bar = 50 μm). Red and yellow colors denote VF and SuF, respectively. (F) The mass of VF and SuF (mm^3^) as measured by micro-CT. (G) Cross-sectional area of EpF (μm^2^) based on H&E staining. All results are expressed as mean ± SD. ^#^*p* < 0.05, ^##^*p* < 0.01, ^###^*p* < 0.001 vs. NC; ^*^p < 0.05,^ **^p < 0.01, ^***^p < 0.001 vs. HC. NC, normal-fat diet control; HC, high-fat diet (HFD) control; PC, HFD with quercetin; FL, HFD with low-dose Fucoidan P; FM, HFD with medium-dose Fucoidan P; FH, HFD with high-dose Fucoidan P; FER, food efficiency ratio; TG, triglyceride; TC, total cholesterol; HDL-C, high-density lipoprotein cholesterol; LDL-C, low-density lipoprotein cholesterol; EpF, epididymal fat; ReF, retroperitoneal fat; SuF, subcutaneous fat; VF, visceral fat.

To assess the effects of Fucoidan P on SO, mice were fed an HFD for eight weeks and then orally administered various concentrations of Fucoidan P for six weeks. Both body weight at 15 weeks and body weight gain were significantly increased in the HC group compared with the NC group. These increases were markedly attenuated in the Fucoidan P-treated groups (Fig. 1A). Compared with the NC group, the HC group exhibited a significant increase in food efficiency ratio (FER). In contrast, no significant difference in FER was observed between the HC and Fucoidan P-treated groups. Serum TC, LDL-C, and ALT levels were significantly elevated by HFD feeding, and these elevations were reversed following treatment with Fucoidan P (Fig. 1B and Fig. S2). Next, the effects of Fucoidan P on body fat were explored. Administration of Fucoidan P reduced the size and weight of white fat in HFD-fed mice, including EpF, ReF, and SuF (Fig. 1C, D).

Micro-CT analysis revealed significantly increased VF (red) and SuF (yellow) in the HC group, whereas a noticeable reduction was observed in the Fucoidan P-treated groups (Fig. 1E, F). H&E staining showed that EpF size was markedly increased as a result of HFD feeding, but Fucoidan P treatment notably reduced this enlargement (Fig. 1E, G). Furthermore, Fucoidan P treatment upregulated several fat browning-related genes, including uncoupling protein 1 (UCP1), PR domain containing 16 (PRDM16), and cell death-inducing DNA fragmentation factor alpha-like effector A (CIDEA), in SuF (Fig. S3). These results indicate that Fucoidan P suppresses obesity in HFD-fed mice.

### Fucoidan P suppresses muscle atrophy in HFD-fed mice

**Figure 2:**
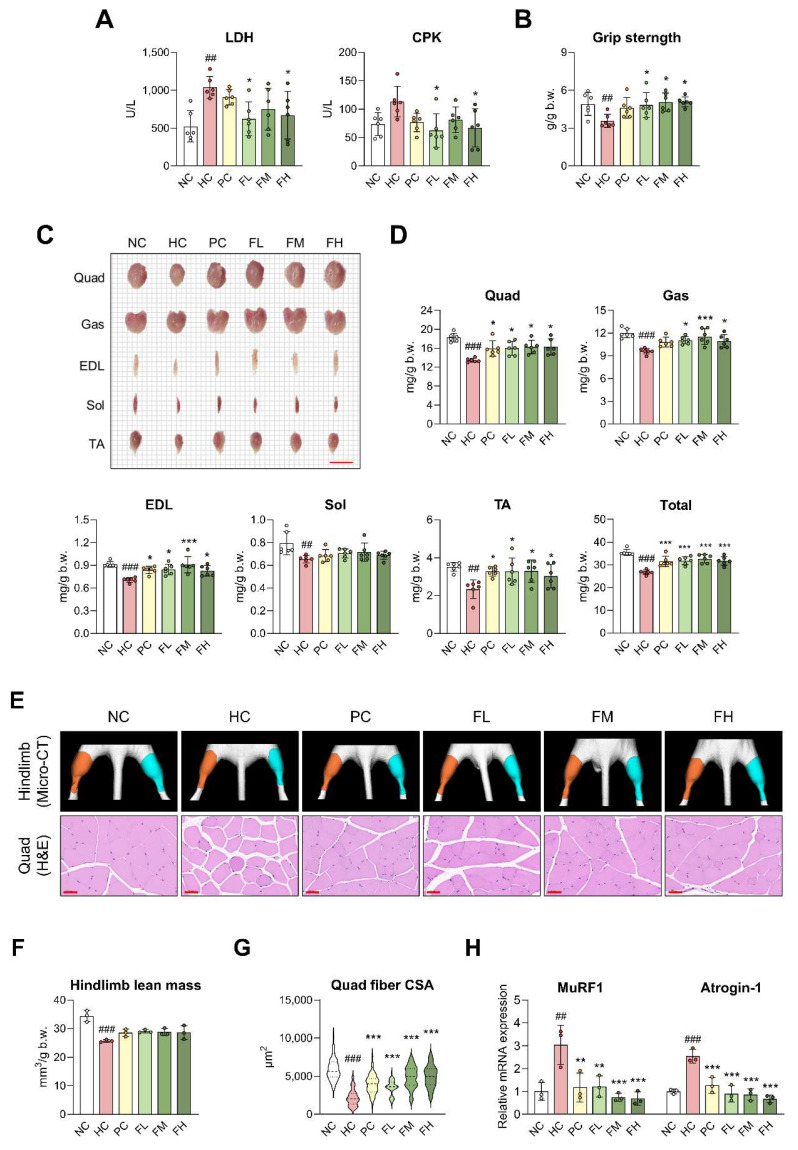
** Effects of Fucoidan P on muscle atrophy in HFD-fed mice.** The mice were fed an HFD for 8 weeks, and then orally administered Fucoidan P or quercetin or saline for 6 weeks. (A) Serum levels of LDH and CPK. (B) Grip strength (g/g b.w.). (C) Representative images of skeletal muscles (scale bar = 1cm). (D) Skeletal muscle weight (mg/g b.w.). Total indicates total skeletal muscle mass calculated as the sum of the weights of Quad, Gas, EDL, Sol, and TA. (E) Representative micro-CT images of hindlimb and representative H&E-stained images of Quad (scale bar = 50 μm). Orange and cyan colors denote left and right hindlimbs, respectively. (F) Hindlimb lean mass (mm^3^/g b.w.) as measured by micro-CT. (G) Cross-sectional area of Quad fiber (μm^2^) based on H&E staining. (H) The relative mRNA expression levels of muscle atrophy-related markers in Quad. All results are expressed as mean ± SD. ^##^*p* < 0.01, ^###^*p* < 0.001 vs. NC; ^*^p < 0.05,^ **^p < 0.01, ^***^p < 0.001 vs. HC. NC, normal-fat diet control; HC, high-fat diet (HFD) control; PC, HFD with quercetin; FL, HFD with low-dose Fucoidan P; FM, HFD with medium-dose Fucoidan P; FH, HFD with high-dose Fucoidan P; LDH, lactate dehydrogenase; CPK, creatine phosphokinase; Quad, quadriceps femoris muscle; Gas, gastrocnemius muscle; EDL, extensor digitorum longus muscle; Sol, soleus muscle; TA, tibialis anterior muscle.

We further examined whether Fucoidan P improved muscle atrophy induced by HFD. Fucoidan P groups exhibited lower serum levels of LDH and CPK compared to the HC group (Fig. 2A). Grip strength was weakened in the HC group but recovered with Fucoidan P administration (Fig. 2B). The size and weight of skeletal muscle were significantly reduced following HFD feeding (Fig. 2C, D). Fucoidan P administration significantly increased the size and weight of most skeletal muscles, except for the Sol. Notably, when evaluated as total skeletal muscle mass, Fucoidan P-treated groups resulted in a highly significant increase compared to the HC group. Micro-CT analysis of the hindlimb revealed a reduction in muscle volume in the HC group compared with the NC group, accompanied by a significant decrease in hindlimb lean mass (Fig. 2E, F). Fucoidan P-treated groups showed a tendency toward increased hindlimb lean mass compared with the HC group. Regarding pathological changes in the Quad, muscle fibers atrophied, became round, and showed decreased fiber CSA due to HFD intake (Fig. 2E, G). However, Fucoidan P administration alleviated pathological changes and increased fiber CSA, a trend that was also observed in Gas (Fig. S4A). Furthermore, Fucoidan P administration markedly downregulated the key muscle atrophy markers, muscle RING-finger protein-1 (MuRF1) and Atrogin-1, in both the Quad (Fig. 2H) and Gas (Fig. S4B).

In addition to muscle atrophy, excessive extracellular matrix (ECM) remodeling is a critical pathological feature associated with obesity-induced muscle dysfunction 21. Therefore, we further investigated the expression of ECM remodeling-related genes in skeletal muscle. HFD feeding significantly upregulated the expression of fibrotic markers, including collagen type I alpha 1 chain (COL1A1) and collagen type III alpha 1 chain (COL3A1), whereas these elevations were markedly suppressed by Fucoidan P administration (Fig. S5A, B). Furthermore, Fucoidan P also reduced the HFD-induced upregulation of matrix metallopeptidase 9 (MMP9) and tissue inhibitor of metalloproteinases 1 (TIMP1) (Fig. S5C, D), suggesting an improvement in the imbalance of ECM remodeling. Collectively, these results indicate that Fucoidan P alleviates muscle atrophy in HFD-fed mice and contributes to the maintenance of ECM homeostasis in skeletal muscle.

### Fucoidan P regulates muscle protein synthesis and degradation via the Akt signaling pathway in skeletal muscle

**Figure 3:**
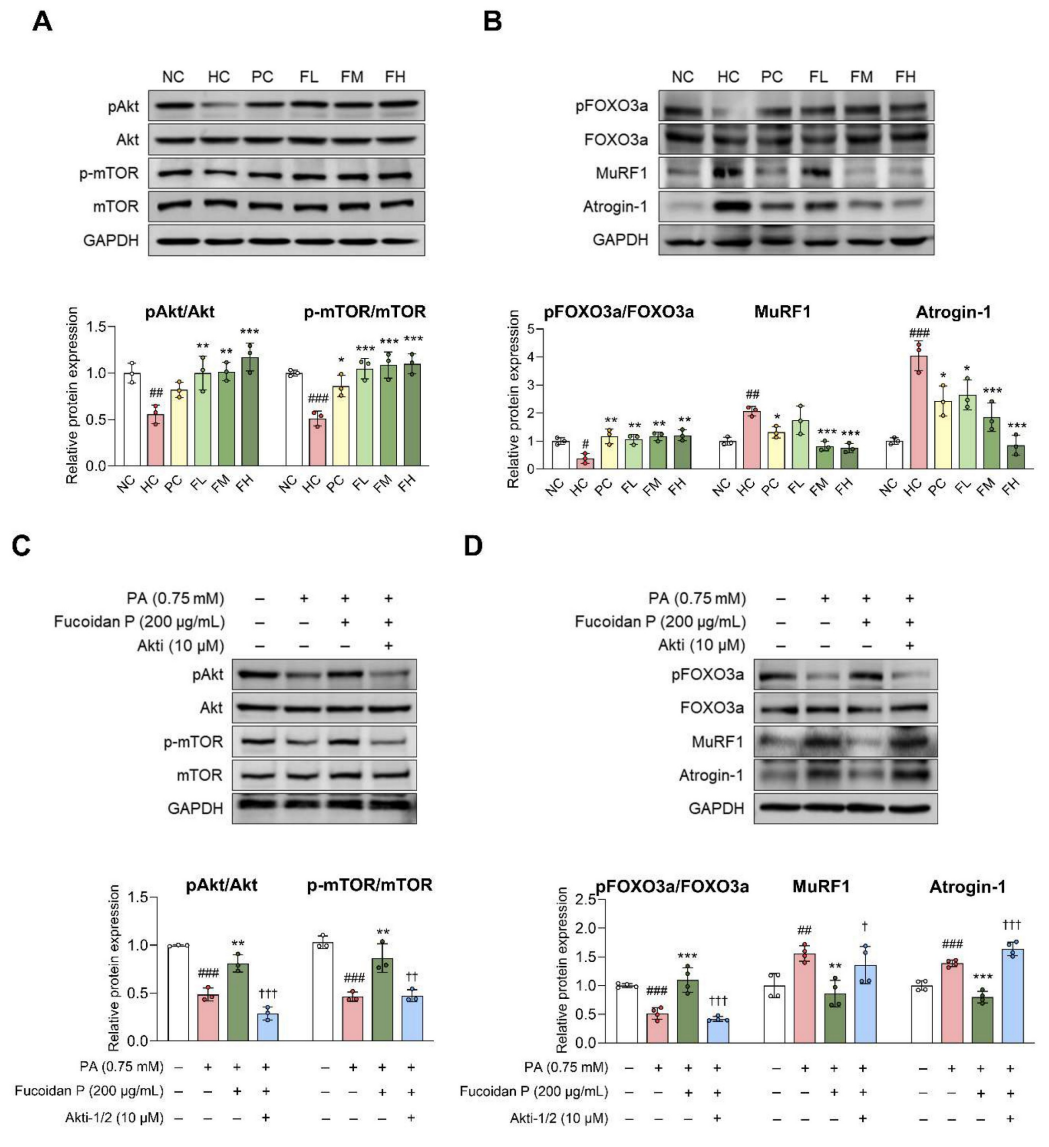
**Effects of Fucoidan P on muscle protein synthesis and degradation via the Akt pathway.** (A, B) The mice were fed an HFD for 8 weeks, and then orally administered Fucoidan P or quercetin or saline for 6 weeks. Relative protein expression levels of (A) protein synthesis-related markers and (B) protein degradation-related markers in Quad. All results are expressed as mean ± SD. ^#^*p* < 0.05, ^##^*p* < 0.01, ^###^*p* < 0.001 vs. NC; ^*^p < 0.05,^ **^p < 0.01, ^***^p < 0.001 vs. HC. (C, D) After myogenic differentiation, C2C12 myotubes were co-treated Fucoidan P and PA with or without Akti-1/2 (Akt inhibitor) for 24 h. Relative protein expression levels of (C) protein synthesis-related markers and (D) protein degradation-related markers in C2C12 myotubes. All results are expressed as mean ± SD. ^##^*p* < 0.01, ^###^*p* < 0.001 vs. vehicle control; ^*^p < 0.05,^ **^p < 0.01,^ ***^p < 0.001 vs. PA; ^†^*p* < 0.05, ^††^*p* < 0.01, ^†††^*p* < 0.001 vs. Fucoidan P. NC, normal-fat diet control; HC, high-fat diet (HFD) control; PC, HFD with quercetin; FL, HFD with low-dose Fucoidan P; FM, HFD with medium-dose Fucoidan P; FH, HFD with high-dose Fucoidan P; Quad, quadriceps femoris muscle; PA, palmitate.

The balance between muscle protein synthesis and degradation is important for maintaining proper body function 8. In the HC group, Akt and mTOR phosphorylation was markedly decreased compared to the NC group (Fig. 3A). Fucoidan P administration significantly increased their phosphorylation levels. HFD also reduced FOXO3a phosphorylation, leading to increased MuRF1 and Atrogin-1 expression, whereas Fucoidan P counteracted these changes in a dose-dependent manner (Fig. 3B). Additionally, Fucoidan P increased phosphorylation of Akt and mTOR, eukaryotic translation initiation factor 4E-binding protein 1 (4E-BP1), and ribosomal protein S6 kinase B1 (S6K), while increasing FOXO3a phosphorylation and inhibiting MuRF1 and Atrogin-1 expression in PA-treated C2C12 myotubes (Fig. S6). To confirm Akt activation by Fucoidan P in this regulation, we treated PA-treated C2C12 myotubes with Fucoidan P and the Akt inhibitor Akti-1/2. Akti-1/2 eliminated the regulatory effect of Fucoidan P on the phosphorylation of Akt, mTOR, and FOXO3a, as well as on the protein expression of MuRF1 and Atrogin-1 (Fig. 3C, D). These results indicate that Fucoidan P regulates the balance between muscle protein synthesis and degradation by activating the Akt signaling pathway.

### Fucoidan P improves energy metabolism via the AMPK/PGC-1α signaling pathway in skeletal muscle

**Figure 4:**
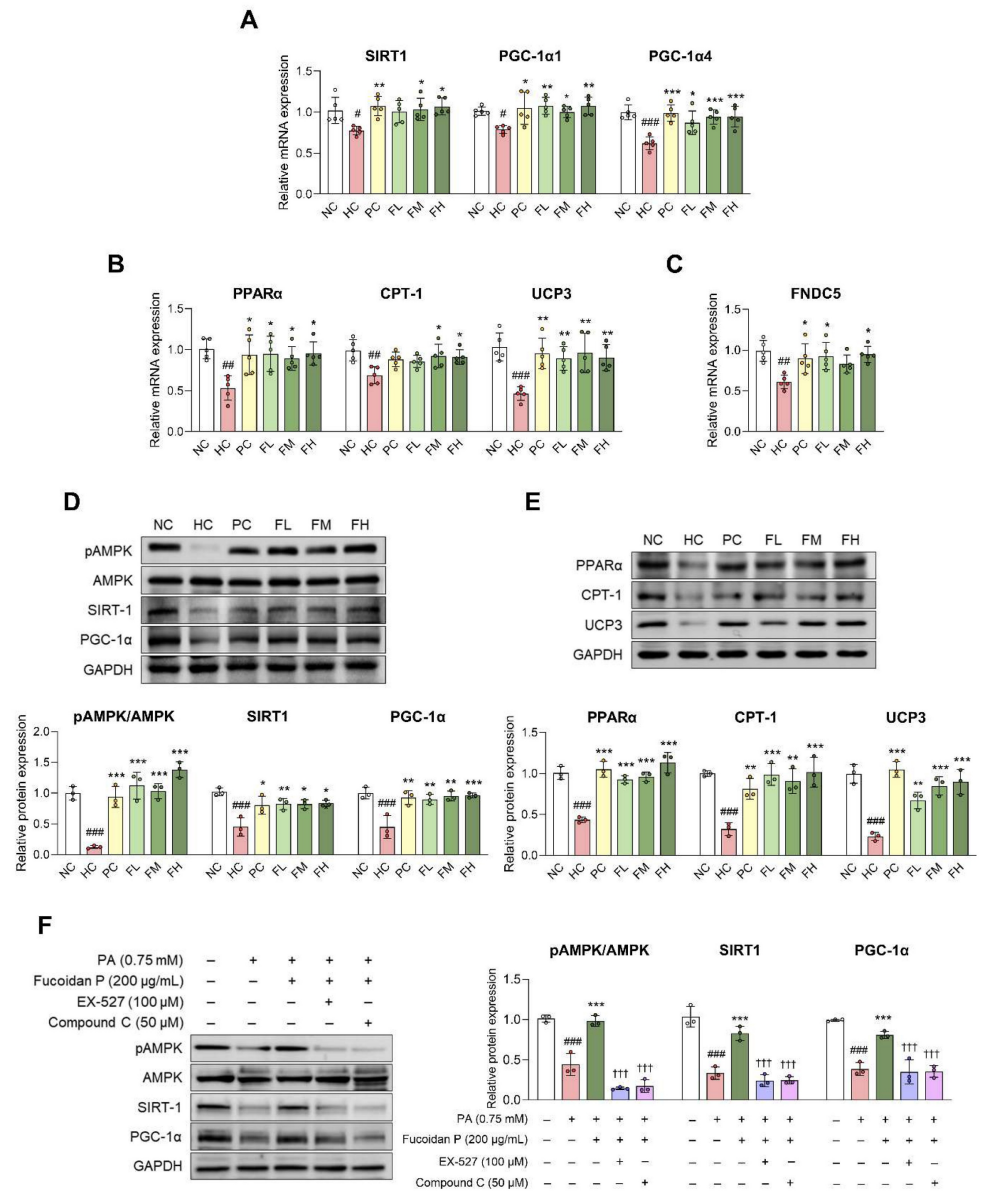
**Effects of Fucoidan P on energy metabolism via the AMPK/SIRT1/PGC-1α pathway.** (A-E) The mice were fed an HFD for 8 weeks, and then orally administered Fucoidan P or quercetin or saline for 6 weeks. The relative mRNA expression levels of (A) mitochondrial biogenesis-related markers, (B) fatty acid oxidation-related markers, and (C) FNDC5 in Quad. (D, E) The relative protein expression levels of energy metabolism-related markers in Quad. All results are expressed as mean ± SD. ^#^*p* < 0.05, ^##^*p* < 0.01, ^###^*p* < 0.001 vs. NC; ^*^p < 0.05,^ **^p < 0.01, ^***^p < 0.001 vs. HC. (F) After myogenic differentiation, C2C12 myotubes were co-treated Fucoidan P and PA or with/without EX-527 (SIRT1 inhibitor) or Compound C (AMPK inhibitor) for 24 h. The relative protein expression levels of energy metabolism-related markers in C2C12 myotubes. All results are expressed as mean ± SD. ^###^*p* < 0.001 vs. vehicle control; ^***^p < 0.001 vs. PA; ^†††^*p* < 0.001 vs. Fucoidan P. NC, normal-fat diet control; HC, high-fat diet (HFD) control; PC, HFD with quercetin; FL, HFD with low-dose Fucoidan P; FM, HFD with medium-dose Fucoidan P; FH, HFD with high-dose Fucoidan P; Quad, quadriceps femoris muscle; PA, palmitate.

AMPK/PGC-1α is a regulator of various biological functions in skeletal muscles, and their activation plays a key role in mitochondrial biogenesis and fatty acid oxidation 7. In this study, Fucoidan P administration significantly increased the mRNA expression levels of mitochondrial biogenesis-related markers SIRT1, PGC-1α1, and PGC-1α4, as well as fatty acid oxidation-related markers PPARα, carnitine palmitoyltransferase 1 (CPT-1), and UCP3 in both the Quad (Fig. 4A, B) and Gas (Fig. S4C, D), compared to the HC group. Additionally, we found that the mRNA expression level of fibronectin type III domain-containing protein 5 (FNDC5) was increased by Fucoidan P administration in both the Quad (Fig. 4C) and Gas (Fig. S4E).

At the protein level, AMPK phosphorylation was significantly reduced following HFD feeding, while it was increased by Fucoidan P administration (Fig. 4D). In addition, the expression levels of SIRT1, PGC-1α, PPARα, CPT-1, and UCP3 were markedly elevated by Fucoidan P treatment (Fig. 4D, E). Moreover, Fucoidan P treatment activated the AMPK/SIRT1/PGC-1α pathway in PA-treated C2C12 myotubes (Fig. S7). To assess whether Fucoidan P regulates energy metabolism via the AMPK signaling pathway, C2C12 myotubes were treated with PA and Fucoidan P, along with either EX-527 (SIRT1 inhibitor) or Compound C (AMPK inhibitor) (Fig. 4F). Both inhibitors abolished the effects of Fucoidan P on AMPK phosphorylation and concurrently suppressed the SIRT1 and PGC-1α expression. These results suggest that Fucoidan P regulates energy metabolism by activating the AMPK/PGC-1α signaling pathway.

### Identification of differentially expressed genes in myotubes from the participants with obesity and validation in HFD-induced SO mice model

**Figure 5:**
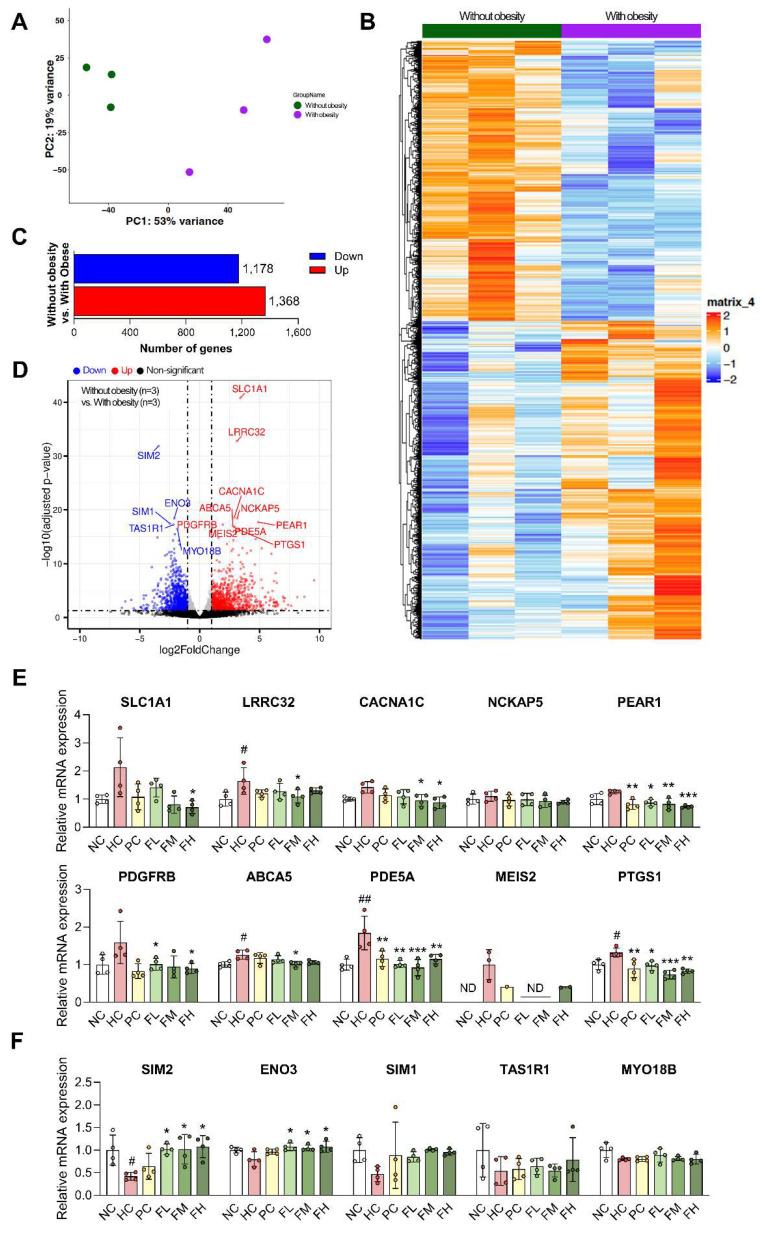
** Differentially expressed gene analysis between obese and non-obese individuals and validation using HFD-induced SO mice model.** (A) Principal component analysis (PCA) of obese and non-obese (control) groups using all genes (n = 14,952). (B) Hierarchical clustering heatmap of the top 5,000 differentially expressed genes (DEGs) between control and obese groups, ranked by ascending adjusted p-value. (C) The 1,178 down-regulated and 1,368 up-regulated in obese group (adjusted p-value < 0.05). (D) Volcano plot displaying DEGs between control group versus obese group. The top 15 DEGs were identified based on adjusted p-values. (E, F) The mRNA expression levels of DEGs in Quad of HFD-fed mice: (E) 10 up-regulated genes and (F) 5 down-regulated genes. All results are expressed as mean ± SD. ^#^*p* < 0.05, ^##^*p* < 0.01 vs. NC; ^*^p < 0.05,^ **^p < 0.01, ^***^p < 0.001 vs. HC. NC, normal-fat diet control; HC, high-fat diet (HFD) control; PC, HFD with quercetin; FL, HFD with low-dose Fucoidan P; FM, HFD with medium-dose Fucoidan P; FH, HFD with high-dose Fucoidan P; Quad, quadriceps femoris muscle.

To explore the translational relevance of our results, we compared our results with muscle gene expression changes observed in transcriptomic datasets GSE63887 (Participants without obesity group) and GSE81965 (Participants with obesity). Skeletal muscle precursor cells isolated from participants without obesity and participants with obesity were differentiated *in vitro* under the same conditions 22. Gene expression patterns in muscle were substantially altered in the participants with obesity compared to the participants without obesity, suggesting that obesity profoundly impacts gene regulation (Fig. 5A, B). DEG analysis identified a total of 2,546 DEGs using a threshold of adjusted p-value ≤ 0.05, comprising 1,368 up-regulated and 1,178 down-regulated genes (Fig. 5C). Among these, the top 15 DEGs, ranked by adjusted p-values, included 10 up-regulated (SLC1A1, LRRC32, CACNA1C, NCKAP5, PEAR1, PDGFRB, ABCA5, PDE5A, MEIS2, and PTGS1) and 5 down-regulated (SIM2, ENO3, SIM1, TAS1R1, and MYO18B) genes (Fig. 5D and Table S3, 4). We performed qRT-PCR on the skeletal muscle of HFD-induced SO mice to validate whether the expression changes of DEG identified in clinical studies were also observed *in vivo*, and the results were consistent with the DEG analysis (Fig. 5E, F). Furthermore, Fucoidan P treatment significantly altered the expression of several DEG, notably downregulating PDE5A and PTGS1, which are linked to inflammation in all Fucoidan P-treated groups.

### Fucoidan P suppresses inflammatory response via the NF-κB inactivation in skeletal muscle

**Figure 6:**
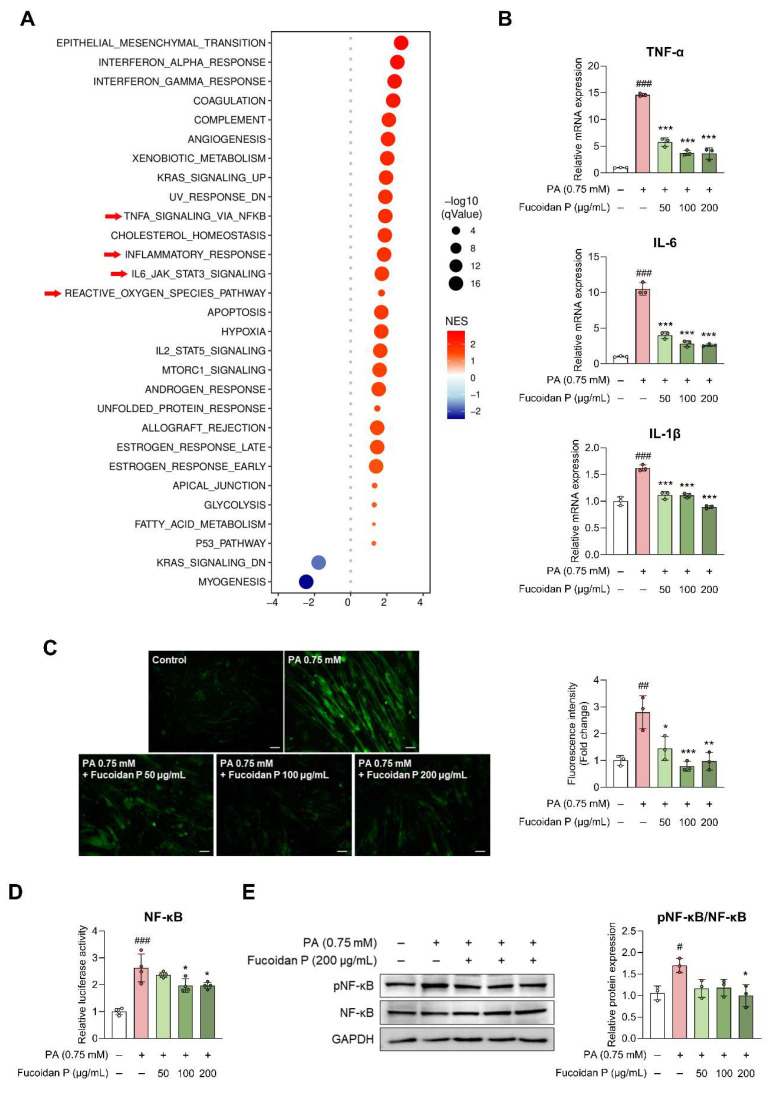
** Effects of Fucoidan P on inflammatory response via the NF-κB pathway.** (A) Bubble plot of significantly enriched hallmark pathways identified by Weighted Concept Signature Enrichment Analysis (WCSEA) in the obese group (FDR < 0.05). Bubble size represents the normalized enrichment score (NES) and bubble color represents false discovery rate (FDR). (B) The relative mRNA expression levels of pro-inflammatory cytokine in PA-treated C2C12. (C) Representative fluorescent images of reactive oxygen species (ROS) and quantified value of fluorescence intensity (scale bar = 200 μm). (D) The relative luciferase activity of NF-κB. (E) The relative protein expression level of pNF-κB/NF-κB. All results are expressed as mean ± SD. ^#^*p* < 0.05, ^##^*p* < 0.01, ^###^*p* < 0.001 vs. vehicle control; ^*^p < 0.05,^ **^p < 0.01, ^***^p < 0.001 vs. PA. PA, palmitate.

The DEG validation results revealed the possibility that Fucoidan P was related to the inflammatory response in SO model mice. To further investigate whether these gene-level changes reflect coordinated alterations at the pathway level, we performed WCSEA using Hallmark gene sets from MSigDB. The analysis revealed significant enrichment of inflammation-related pathways in the participants with obesity, including TNFA_SIGNALING_VIA_NFKB, INFLAMMATORY_RESPONSE, IL6_JAK_STAT3_SIGNALING, and REACTIVE_OXYGEN_SPECIES_PATHWAY (Fig. 6A). These results were further validated *in vitro*. PA treatment upregulated pro-inflammatory cytokines, including TNF-α, IL-6, and IL-1β, in C2C12 myotubes, and this effect was attenuated by Fucoidan P in a dose-dependent manner (Fig. 6B). This was consistent with the *in vivo* results, except for serum TNF-α (Fig. S8A, B). ROS were elevated by PA treatment in C2C12 myotubes, but were significantly diminished following Fucoidan P treatment (Fig. 6C). Furthermore, Fucoidan P suppressed nuclear factor-kappa B (NF-κB) activation in PA-treated C2C12 myotubes, as evidenced by reduced luciferase activity and inhibition of NF-κB phosphorylation (Fig. 6D, E). Similarly, comparable anti-inflammatory effects were observed in vivo (Fig. S8C), suggesting that Fucoidan P alleviates inflammatory response by targeting the NF-κB pathway. In addition, Fucoidan P markedly reduced the phosphorylation levels of mitogen-activated protein kinase (MAPK) and the activation of activator protein-1 (AP-1), which are key mediators of inflammation. (Fig. S9).

### Fucoidan P regulates transcription factor target genes identified through clinical RNA-seq in PA-treated C2C12 myotubes

To further understand the transcriptional regulatory mechanisms underlying obesity-induced muscle dysfunction, we conducted transcription factor (TF) activity analysis. TF activity in the participants with obesity was remarkedly distinct from that in the participants without obesity, indicating that obesity changes the transcriptional regulatory network (Fig. S10A). We found that 12 TFs (MEF2D_MEF2A, MYOD1, MEF2C, MEF2B, TFAP4_MSC, HBP1, RBPJ, SOX3_SOX2, TCF3_MYOG, ID4_TCF4_SNAI2, PKNOX1_TGIF2, and HOXA5) had higher activities in the participants without obesity than participants with obesity (Fig. S10B). On the other hand, 18 TFs (CEBPE_CEBPD, IRF2_STAT2_IRF8_IRF1, CUX2, FOXL1, ATF4, ZBTB18, CEBPB, CREB3L2, NFATC2_NFATC3, HIF1A, ARNT, STAT1_STAT3_BCL6, T, OTX1, VAX2_RHOXF2, TLX1_NFIC, TP63, and AR_NR3C2) had activities in the participants with obesity than the participants without obesity. Subsequently, the target genes of MEF2D_MEF2A, the TF that showed the largest absolute mean difference, were validated using qRT-PCR analysis in C2C12 myotubes treated with PA (Fig. S10C-E). As predicted by the TF activity analysis, MEF2D and MEF2A target genes were significantly downregulated following PA treatment, including myoblast determination protein 1 (MyoD), myosin heavy chain (Myh) isoforms (Myh1, Myh2a, Myh2b, and Myh2x), and PGC-1α isoforms (PGC-1α1, PGC-1α2, PGC-1α3, and PGC-1α4). Notably, Fucoidan P treatment restored the expression of MyoD, Myh2 isoforms, and PGC-1α isoforms, which had been suppressed by PA. These findings suggest that PA diminished MEF2D and MEF2A target gene expression, which Fucoidan P could potentially counteract.

## Discussion

SO is closely associated with a wider range of complex health issues than obesity or sarcopenia alone 23. Although several agents have been proposed to prevent and improve SO, no specific therapeutic agents have yet been clearly established. Furthermore, to the best of our knowledge, the effects of Fucoidan P on SO have not been previously reported. This study investigated how Fucoidan P influences SO alleviation and its associated molecular pathways. The main findings of this study are as follows: (i) Fucoidan P suppressed obesity and muscle atrophy in HFD-fed mice. (ii) Fucoidan P regulated muscle protein synthesis and degradation through the Akt signaling pathway. (iii) Fucoidan P enhanced energy metabolism through the AMPK/PGC-1α signaling pathway. (iv) Fucoidan P inhibited the inflammatory response through the suppression of NF-κB activation.

Obesity plays a central role in the progression of SO by exacerbating skeletal muscle dysfunction. Accordingly, we first examined the anti-obesity effects of Fucoidan P in HFD-fed mice. Fucoidan P attenuated HFD-induced body weight gain without affecting FER values and reduced overall fat mass, accompanied by improvements in serum lipid profiles. These findings suggest that Fucoidan P alleviates obesity-associated metabolic disturbances under HFD conditions.

Obesity-induced fat overload is known to trigger inflammatory responses, metabolic disorders, and mitochondrial dysfunction within skeletal muscles, ultimately promoting muscle atrophy 24, 25. These changes are linked to impaired intracellular signaling pathways involved in the maintenance of muscle homeostasis. In this study, HFD feeding exacerbated muscle atrophy by decreasing muscle mass and strength and, reducing muscle fiber CSA, which were accompanied by impaired Akt signaling. Akt signaling plays a key role in regulating muscle homeostasis by coordinating protein synthesis and degradation in both cellular and animal models. In skeletal muscle, activated Akt phosphorylates mTOR and FOXO, promoting protein synthesis and inhibiting protein degradation 8. In individuals with sarcopenia, the mTOR signaling pathway is often impaired, and inhibition of this pathway has been shown to adversely impact skeletal growth 26. Furthermore, under muscle wasting conditions like sarcopenia, diminished Akt activity facilitates the nuclear localization of FOXO. This process activates muscle-specific ubiquitin ligases, including MuRF1 and Atrogin-1 27. Fucoidan P attenuated HFD-induced muscle atrophy and enhanced Akt signaling, with increased phosphorylation of mTOR and FOXO3a. Especially, these effects were blocked by Akti-1/2, suggesting that Fucoidan P mitigates muscle atrophy by modulating the balance between protein synthesis and degradation through Akt-mediated downstream signaling.

In addition to muscle-intrinsic signaling pathways, the muscle microenvironment has also been implicated in the progression of muscle atrophy. Dysregulation of ECM homeostasis is associated with reduced muscle mass and decreased muscle fiber CSA, and excessive lipid supply induces ECM remodeling characterized by collagen accumulation and metabolic inflexibility in the skeletal muscle 28, 29. We observed that HFD feeding significantly increased the expression of COL1A1, COL3A1, MMP9, and TIMP1, consistent with previous studies in individuals with obesity and type 2 diabetes 28, 30. Notably, Fucoidan P partially normalized this imbalance, thereby suggesting that its protective effects against HFD-induced muscle atrophy may involve both muscle-intrinsic signaling and extrinsic mechanisms.

As maintaining the balance between protein synthesis and degradation requires substantial cellular energy, adequate cellular energy availability is essential to support the restoration of muscle metabolic function promoted by Akt signaling 31. In this study, Fucoidan P-mediated improvement of energy metabolism, including AMPK pathway, may help sustain the anabolic responses associated with Akt signaling. AMPK is a key kinase that regulates energy metabolism, including mitochondrial biogenesis and fatty acid oxidation in skeletal muscles 32. As mitochondria are the primary source of ATP required to meet the high energy demands of skeletal muscle, proper regulation of mitochondrial energy metabolism is essential for muscle homeostasis. 33-35. In C2C12 myotubes, AMPK activation increases intracellular NAD^+^ levels, which subsequently activates SIRT1, leading to deacetylation and activation of PGC-1α 36-38. Particularly, PGC-1α exists in multiple isoforms, including PGC-1α1, PGC-1α2, PGC-1α3, and PGC-1α4, each with distinct functional roles in different tissues. In skeletal muscle, PGC-1α1 primarily regulates mitochondrial biogenesis, insulin sensitivity, and muscle fiber formation and maintenance, while PGC-1α4 contributes to muscle hypertrophy through its regulation of IGF-1 and suppression of myostatin 39, 40. Our results demonstrated that Fucoidan P significantly altered PGC-1α1 and PGC-1α4 expression, suggesting potential involvement in the regulation of protein and energy metabolic processes in skeletal muscle. PGC-1α also promotes FNDC5 expression in skeletal muscle, which is subsequently cleaved into circulating irisin 41. Irisin, a myokine, is transported through the bloodstream to various tissues, where it regulates metabolic activities and promotes the browning of white fat 41. Boström et al. demonstrated an upregulation of UCP1, PRDM16, and CIDEA in SuF, along with increased FNDC5 expression in skeletal muscle of PGC-1-α transgenic mice 37. These observations are consistent with our results. Additionally, a previous study demonstrated that HFD-induced changes in FNDC5 expression in skeletal muscles were positively correlated with circulating irisin levels 42. These findings support our suggestion that Fucoidan P may contribute to white fat browning and influences metabolic processes involved in energy utilization within skeletal muscles via the PGC-1α/FNDC5/irisin pathway. However, additional research is required to elucidate the precise mechanisms and potential interactions between fat browning and SO.

Considering that Fucoidan P is a high-molecular-weight polysaccharide, its biological effects on skeletal muscle are likely mediated through indirect or systemic mechanisms, such as modulation of metabolic mediators or involvement of the gut microbiota-muscle axis 43-45. These upstream regulatory processes may subsequently influence intracellular signaling pathways, including Akt and AMPK signaling, observed in skeletal muscle.

While we have elucidated the molecular pathways of Fucoidan P in alleviating SO using animal and cellular models, there are limitations in translating these findings to human clinical outcomes. Therefore, we conducted additional studies utilizing open-source data to evaluate the potential clinical relevance of these findings. In this study, transcriptome analysis revealed that myotubes derived from skeletal muscle progenitor cells obtained from the participants with obesity were linked to genes and pathways involved in inflammatory response. Obesity, which induces persistent mild systemic inflammation, contributes to skeletal muscle inflammation and metabolic disorders, such as muscle protein catabolism, ultimately leading to conditions like sarcopenia 46. Based on DEG analysis results, PDE5A and PTGS1, both associated with the inflammatory response, were significantly increased in the participants with obesity, and similar upregulations were also observed in our HFD-feeding animal model. PDE5A regulates cGMP signaling and has been implicated in inflammatory processes 47, 48, while PTGS1 (COX-1) promotes inflammatory responses 49. Notably, previous studies have shown that inhibition of PDE5A reduces inflammatory markers (TNF-α, IL-6, and IL-1β) 47, 48, 50, and inhibition or genetic deletion of PTGS1 attenuates NF-κB-mediated inflammation 51. In line with these observations, our study demonstrated that Fucoidan P significantly downregulated PDE5A and PTGS1, suggesting that Fucoidan P may have potential clinical relevance in SO.

In addition, PDE5A overexpression has been reported to attenuate Akt phosphorylation in C2C12 myotubes 52, suggesting that PDE5A may be also involved in metabolic signaling pathways relevant to skeletal muscle homeostasis. In this study, PDE5A expression was elevated under HFD conditions, whereas Fucoidan P administration reduced PDE5A expression along with increased phosphorylation of Akt/mTOR signaling. However, further studies are required to clarify the precise role of Fucoidan P in modulating the PDE5A-Akt signaling.

Although each signaling pathway was examined individually, increasing evidence suggests that Akt, AMPK/PGC-1α, and NF-κB pathways may engage in potential crosstalk to cooperatively regulate skeletal muscle inflammation and metabolic homeostasis. SIRT1, which is indirectly activated by AMPK, has been reported to enhance Akt phosphorylation and deacetylate FOXO3a, preventing the induction muscle-specific ubiquitin ligases 53. PGC-1α also negatively regulates FOXO-mediated ubiquitin ligases involved in muscle protein degradation 54. Furthermore, AMPK has been reported to indirectly suppress NF-κB signaling through multiple downstream mediators, including SIRT1 and PGC-1α, thereby attenuating the expression of pro-inflammatory genes 55. These findings suggest that Fucoidan P exerts its beneficial effects on SO through multiple interconnected signaling pathways.

## Conclusion

This study demonstrates that Fucoidan P regulates a multi-pathway network involving muscle protein synthesis and degradation, and energy metabolism through the activation of Akt and AMPK/PGC-1α signaling pathways. Furthermore, the identification of PDE5A and PTGS1 through GEO-based transcriptomic analysis adds clinical context to the anti-inflammatory effects observed in our experimental models. Consistent with these findings, Fucoidan P suppresses NF-κB signaling, suggesting its potential to mitigate the inflammatory responses associated with SO. Taken together, these findings indicate that Fucoidan P ameliorates SO by modulating a network of interconnected signaling pathways. While our animal experiments demonstrated the potential clinical relevance of Fucoidan P, the limited availability of clinical datasets and the uncertainty regarding its effects in humans remain limitations. Therefore, further studies are necessary to validate its clinical efficacy.

## Supplementary Material

Supplementary method, figures and tables.

## Abbreviations

Sarcopenic obesity: SO
AMP-activated protein kinase: AMPK
peroxisome proliferator-activated receptor gamma coactivator alpha: PGC-1α
mammalian target of rapamycin: mTOR
forkhead box O: FOXO
palmitate: PA
high-fat diet: HFD
gene expression omnibus: GEO
Dulbecco's Modified Eagle's Medium: DMEM
fetal bovine serum: FBS
horse serum: HS
normal-fat diet: NFD
positive control: PC
quadriceps femoris: Quad
gastrocnemius: Gas
extensor digitorum longus: EDL
soleus: Sol
tibialis anterior: TA
epididymal fat: EpF
retroperitoneal fat: ReF
subcutaneous fat: SuF
triglyceride: TG
total cholesterol: TC
high-density lipoprotein cholesterol: HDL-C
aspartate transaminase: AST
alanine aminotransferase: ALT
lactate dehydrogenase: LDH
creatine phosphokinase: CPK
low-density lipoprotein cholesterol: LDL-C
visceral fat: VF
cross-sectional area: CSA
tumor necrosis factor-alpha: TNF-α
interleukin: IL
differentially expressed gene: DEG
principal component analysis: PCA
variance-stabilizing transformation: VST
weighted concept signature enrichment analysis: WCSEA
normalized enrichment score: NES
false discovery rate: FDR
food efficiency ratio: FER
eukaryotic translation initiation factor 4E-binding protein 1: 4E-BP1
ribosomal protein S6 kinase B1: S6K
uncoupling protein: UCP1
PR domain containing 16: PRDM16
cell death-inducing DNA fragmentation factor alpha-like effector A: CIDEA
carnitine palmitoyltransferase 1: CPT-1
fibronectin type III domain-containing protein 5: FNDC5
nuclear factor-kappa B: NF-κB
mitogen-activated protein kinase: MAPK
activator protein-1: AP-1
transcription factor: TF
myoblast determination protein 1: MyoD
myosin heavy chain: Myh
COL1A1: collagen type I alpha 1 chain
COL3A1: collagen type III alpha 1 chain
MMP9: matrix metallopeptidase 9
TIMP1: tissue inhibitor of metalloproteinases 1
