# Supplementary material for: Fucoidan P Alleviates Sarcopenic Obesity by Regulating Muscle Protein and Energy Metabolism
Source: Int J Biol Sci. 2026 Apr 8;22(8):4367–82. doi: 10.7150/ijbs.125462 (PMC13137976; doi:10.7150/ijbs.125462)
Supplement: Supplementary file 1 — Supplementary method, figures and tables. [file ijbsv22p4367s1.pdf]

Supplementary information

Fucoidan P Alleviates Sarcopenic Obesity by Regulating Muscle Protein and Energy Metabolism

Jong-Yeon Kim [1], Sung-Min Kim [1], Sanghoon Lee [2], Ju-Hong Jeon [2], Eun-Jung Park [3,4,*], Hae-Jeung Lee [1,3,4,5,6*]

1 Department of Food Science and Biotechnology, Gachon University, Gyeonggi-do 13120, Republic of Korea

2 Department of Physiology & Biomedical Sciences and Institute of Human-Environment Interface Biology, Seoul National University College of Medicine, Seoul 03080, Republic of Korea

3 Department of Food and Nutrition, Gachon University, Gyeonggi-do 13120, Republic of Korea

4 Institute for Aging and Clinical Nutrition research, Gachon University, Gyeonggi-do 13120, Republic of Korea

5 Department of Health Sciences and Technology, GAIHST, Gachon University, Incheon 21999, Republic of Korea

6 Gachon Biomedical Convergence Institute, Gachon University Gil Medical Center, Incheon 21565, Republic of Korea

* Correspondence authors.

E-mail addresses: skysea@gachon.ac.kr, skysea1010@gmail.com (Hae-Jeung Lee), ejpark@gachon.ac.kr (Eun-Jung Park)

Contents:

Supplementary Method

Supplementary Tables 1 to 4

Supplementary Figures 1 to 10

Supplementary Reference

Supplementary Method

Transcription factor (TF) activities in the Obesity

Transcriptomic data of obese were analyzed using Integrated System for Motif Activity Response Analysis (ISMARA, https://ismara.unibas.ch/mara/) [S1]. This tool can infer regulatory networks from gene expression, calculate TF motif activities, and identify the key TFs driving the observed expression state changes. ISMAMRA predicted activities of the regulators across the samples, their genome-wide targets, enriched gene categories among the targets, and direct interactions between the regulators. Subsequently, the resulting P-values (one for each TF) underwent adjustment for multiple hypothesis testing using the false discovery rate (FDR) method. The analytical results were depicted as a volcano plot, with the x-axis representing the mean TF activity difference between the participants without obesity and participants with obesity and the y-axis indicating FDR q-value. Significant TF motifs were selected based on an absolute mean TF activity difference > 0.05.

Supplementary Tables

Table S1. Primer sequences for qRT-PCR analysis

Gene	Primer sequence (5'-3')	Accession number
MuRF1	F: GACAGTCGCATTTCAAAGCA<br>R: AGGGATTCGCAGCCTGGAAG	NM_001039048.2
Atrogin-1	F: CAGCTTCGTGAGCGACCTC<br>R: GGCAGTCGAGAAGTCCAGTC	NM_026346.3
SIRT1	F: TGTGAAGTTACTGCAGGAGTGTAAA<br>R: GCATAGATACCGTCTCTTGATCTGAA	NM_019812.3
PGC-1α1	F: GGACATGTGCAGCCAAGACTCT<br>R: CACTTCAATCCACCCAGAAAGCT	NM_008904.3
PGC-1α2	F: CCACCAGAATGAGTGACATGGA<br>R: GTTCAGCAAGATCTGGGCAAA	JX866946.1
PGC-1α3	F: AAGTGAGTAACCGGAGGCATTC<br>R: TTCAGGAAGATCTGGGCAAAGA	JX866947.1
PGC-1α4	F: TCACACCAAACCCACAGAAA<br>R: CTGGAAGATATGGCACAT	JX866948.1
PPARα	F: CAGGAGAGCAGGGATTTGCA<br>R: CCTACGCTCAGCCCTCTTCAT	NM_011144.6
CPT-1	F: CTCAGTGGGAGCGACTCTTCA<br>R: GGCCTCTGTGGTACACGACAA	AF017175.1
UCP3	F: ACTCCAGCGTCGCCATCAGGATTCT<br>R: TAAACAGGTGAGACTCCAGCAACTT	NM_009464.3
FNDC5	F: ATGAAGGAGATGGGGAGGAA<br>R: GCGGCAGAAGAGAGCTATAACA	NM_027402.4
UCP1	F: CCTGCCTCTCTCGGAAACAA<br>R: GTAGCGGGGTTTGATCCCAT	NM_009463.3
PRDM16	F: AGAGGATGAGGAACCACCCA<br>R: AAATGCTTCCTCAGCTGCTCT	NM_027504.4
CIDEA	F: ATCACAACTGGCCTGGTTACG<br>R: TACTACCCGGTGTCCATTTCT	NM_007702.2
TNF-α	F: ATGAGCACAGAAAGCATGATC<br>R: TACAGGCTTGTCACTCGAATT	NM_013693.3
IL-6	F: AGTTGCCTTCTTGGGACTGA<br>R: CAGAATTGCCATTGCACAAC	NM_031168.2
IL-1β	F: GGGCCTCAAAGGAAAGAATC<br>R: TACCAGTTGGGGAACTCTGC	XM_006498795.5
COL1A1	F: GCTCCCTTGGACATTGGT<br>R: GGAAAAGTGGGCTGGGT	NM_007742.4
COL3A1	F: AAAGTGAGGGAAGCCAAAC<br>R: TGCAAAAGAGGAGAGAGGA	NM_009930.2
MMP9	F: CGACTTTTGTGGTCTTCCCC<br>R: GACTGCTTCTCTCCCATCATC	NM_013599.5
TIMP1	F: CGAGACCACCTTATACCAGCG<br>R: ATGACTGGGGTGTAGGCGTA	NM_011593.3
SLC1A1	F: GCTGTGCGGAAGAAAAGAAC<br>R: ACGATCTGCCCAATGCTTAG	NM_009199.3
LRRC32	F: GGGTTGCGCGAACGATG<br>R: TTGTTCACCGTCCTACAGGG	NM_001113379.2
CACNA1C	F: TTCACAGCTGAGTGACAGGG<br>R: CTGAACAAAGGCCCGAATCA	XM_036165774.1
NCKAP5	F: CTGTGGAAGCTGAGTGTTGG<br>R: TCCGGGGTTCTGTCTCATTC	NM_001081756.1

PEAR1	F: AGCTGTAATGTGCCCTGTTC<br>R: TGGCAGGGAACAAATGACAC	NM_001032414.1
PDGFRB	F: CTTGCCCTTCAAAGTGGTGG<br>R: GTGGAGTCGTAAGGCAACTG	NM_001146268.1
ABCA5	F: CTTCCTGGCAATCCACATCG<br>R: TTGCGATGACAGCCATAAGC	NM_147219.2
PDE5A	F: GAGAGAGAGAGGTGGAAATCCG<br>R: AGAGAAGGTAAAGTCCCGGTG	NM_153422.3
MEIS2	F: GACATGGACCCAATGCATCA<br>R: TCCGCCAACATTGGGATCTA	U57343.1
PTGS1	F: ATTGCACATCCATCCACTCC<br>R: CTGGTTCTGGCACGGATAGT	XM_011239036.4
SIM2	F: TTAATAGAACTCCGCAGCCG<br>R: ACAGATTGACACCTGGATGC	NM_011377.2
ENO3	F: CAGCTGCTACCTAGAGGAGAC<br>R: GAATCGACCCTTGGCTGTG	NM_001136062.3
SIM1	F: GTTACGCCACCATCGTACAC<br>R: TAGTGGGAGTGGAACTGCTG	NM_011376.3
TAS1R1	F: TGCAGTTATGAAGCGTCTGGG<br>R: TGAAAGCCCAGCAGTAAGC	NM_031867.2
MYO18B	F: CTTGGCCACCAGTGTGTCTG<br>R: CGAATCTTCTGCTCCCACAG	NM_028901.2
MyoD	F: GCCAGAGCTGATCCTTGAGT<br>R: AGGGCTCCAGAAAGTGACAA	NM_010866.2
Myh1	F: ACAAGCTGCAGCTGAAGGTG<br>R: TCATTCAGGCCCTTGGCAC	NM_080728.3
Myh2a	F: CCAGCTGCACCTTCTCGTTTGCCAG<br>R: CATGGGGAAGATCTGGTCTTCTT	NM_001039545.2
Myh2b	F: CCTGGAACAGACAGAGAGGAGCAGGAGAG<br>R: GTGAGTTCCTTCACTCTGCGCTCGTGC	NM_010855.3
Myh2x	F: TGCAACAGTTCTTCAACCAC<br>R: GCCAGGTCCATCCCAAAGT	NM_030679.2
GAPDH	F: AGGTCGGTGTGAACGGATTT<br>R: TGTAGACCATGTAGTTGAGG	NM_001289726.2

MuRF1, muscle RING-finger protein-1; PGC-1α, peroxisome proliferator-activated receptor (PPAR) gamma coactivator alpha; UCP, uncoupling protein; PRDM16, PR domain containing 16; CIDEA, cell death-inducing DNA fragmentation factor alpha-like effector A; SIRT1, sirtuin-1; CPT-1, Carnitine palmitoyltransferase 1; FNDC5, fibronectin type III domain-containing protein 5; TNF-α, tumor necrosis factor-alpha; IL-, interleukin-; COL1A1, collagen type I alpha 1 chain; COL3A1, collagen type III alpha 1 chain; MMP9, matrix metallopeptidase 9; TIMP1, tissue inhibitor of metalloproteinases 1; SLC1A1, solute carrier family 1; LRRC32, leucine rich repeat containing 32; CACNA1C, calcium voltage-gated channel subunit alpha1 C; NCKAP5, NCK associated protein 5; PEAR1, platelet endothelial aggregation receptor 1; PDGFRB, platelet derived growth factor receptor beta; ABCA5, ATP binding cassette subfamily A member 5; PDE5A, phosphodiesterase 5A; MEIS2, Meis homeobox 2; PTGS1, prostaglandin-endoperoxide synthase 1; SIM, SIM bHLH transcription factor; ENO3, enolase 3; TAS1R1, taste 1 receptor member 1; MYO18B, myosin XVIIIb; MyoD, myoblast determination protein 1; Myh, myosin heavy chain; GAPDH, glyceraldehyde-3-phosphate dehydrogenase

Table S2. Primary antibodies for western blot analysis

Antibody	Supplier	Dilution	Source	Molecular weight (kDa)
pFOXO3a	CST	1:1000	Rabbit	97
FOXO3a	CST	1:1000	Rabbit	82-97
MuRF1	Abcam	1:1000	Rabbit	40
Atrogin-1	Abcam	1:1000	Rabbit	42
pAkt	CST	1:1000	Rabbit	60
Akt	CST	1:1000	Rabbit	60
p-mTOR	CST	1:1000	Rabbit	289
mTOR	CST	1:1000	Rabbit	289
p4E-BP1	CST	1:1000	Rabbit	15-20
4E-BP1	CST	1:1000	Rabbit	15-20
pS6K	CST	1:1000	Rabbit	70
S6K	CST	1:1000	Rabbit	70
pAMPK	CST	1:1000	Rabbit	62
AMPK	CST	1:1000	Rabbit	62
SIRT1	CST	1:1000	Rabbit	120
PGC-1α	Bioss	1:1000	Rabbit	88
PPARα	Abcam	1:1000	Rabbit	52
CPT-1	Abcam	1:1000	Mouse	88
UCP3	Abcam	1:1000	Rabbit	33
pNF-κB	CST	1:1000	Rabbit	65
NF-κB	CST	1:1000	Rabbit	65
p-p38	CST	1:1000	Rabbit	43
P38	CST	1:1000	Rabbit	40
pJNK	CST	1:1000	Rabbit	46, 54
JNK	CST	1:1000	Rabbit	46, 54
GAPDH	CST	1:10000	Rabbit	37

FOXO, forkhead box O; MuRF1, muscle RING-finger protein-1; mTOR, mammalian target of rapamycin; 4E-BP1, eukaryotic translation initiation factor 4E (eIF4E)-binding protein 1; S6K, ribosomal protein S6 kinase B1; AMPK, AMP-activated protein kinase; SIRT1, sirtuin-1; PGC-1α, peroxisome proliferator-activated receptor (PPAR) gamma coactivator alpha; CPT-1, Carnitine palmitoyltransferase 1; UCP3, uncoupling protein3; NF-κB, nuclear factor-kappa B; JNK, c-Jun N-terminal kinase; GAPDH, glyceraldehyde-3-phosphate dehydrogenase; CST, Cell Signaling Technology

Table S3. List of up-regulated DEGs from top 15 in participants with obesity

GeneSymbol	Log2FC	lFcSE	stat	pvalue	padj
SLC1A1	3.391	0.239	14.192	1.023E-45	1.530E-41
LRRC32	3.111	0.243	12.779	2.159E-37	1.614E-33
CACNA1C	2.894	0.294	9.848	7.012E-23	2.621E-17
NCKAP5	3.135	0.321	9.763	1.621E-22	4.039E-19
PEAR1	4.882	0.509	9.586	9.170E-22	1.959E-18
PDGFRB	1.929	0.204	9.436	3.881E-21	5.803E-18
ABCA5	2.763	0.295	9.379	6.646E-21	9.034E-18
PDE5A	2.931	0.315	9.310	1.282E-20	1.586E-17
MEIS1	2.851	0.306	9.302	1.379E-20	1.586E-17
PTGS1	3.204	0.349	9.179	4.351E-20	4.337E-17

Table S4. List of down-regulated DEGs from top 15 in participants with obesity

GeneSymbol	Log2FC	IFcSE	stat	pvalue	padj
SIM2	-3.436	0.273	-12.575	2.903E-36	1.477E-32
ENO3	-2.188	0.217	-9.766	1.575E-22	4.039E-19
SIM1	-2.488	0.262	-9.509	1.934E-21	3.614E-18
TAS1R1	-2.180	0.231	-9.444	3.575E-21	5.803E-18
MYO18B	-1.862	0.202	-9.260	3.264E-20	3.486E-17

Supplementary Figures

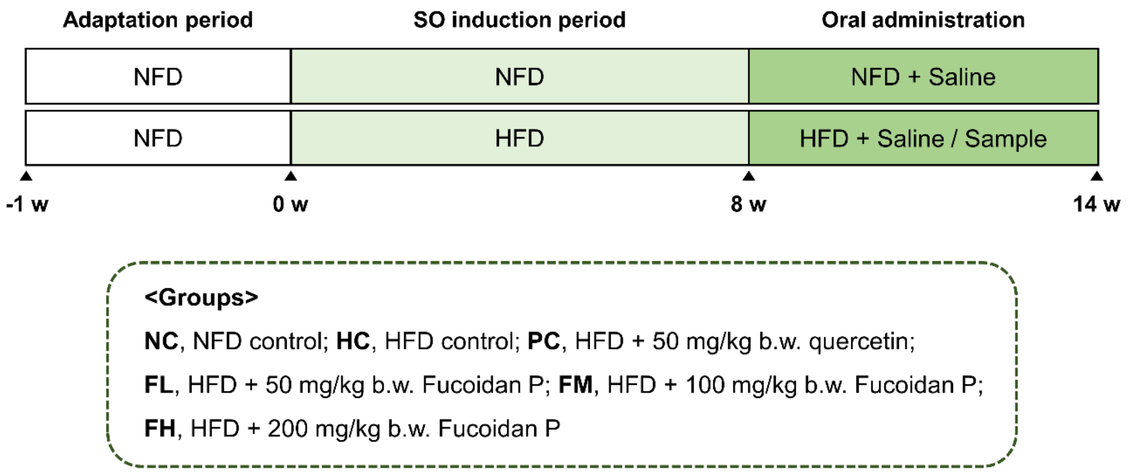

Fig. S1 Schematic representation of the animal experimental schedule. NFD, normal-fat diet; HFD, high-fat diet; PC, positive control

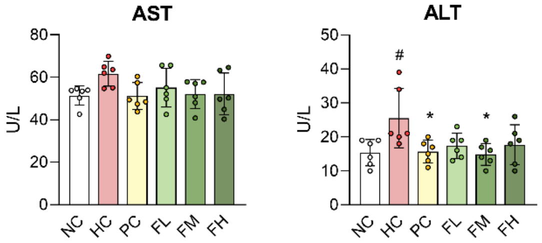

Fig. S2 Effects of Fucoidan P on serum parameters in HFD-fed mice. The mice were fed an HFD for 8 weeks, and then orally administered Fucoidan P or quercetin or saline for 6 weeks. Serum AST and ALT levels were measured using an automated analyzer. All results are expressed as mean ± SD. $^{\#}p < 0.05$ vs. NC; $^{*}p < 0.05$ vs. HC. NC, normal-fat diet control; HC, high-fat diet (HFD) control; PC, HFD with quercetin; FL, HFD with low-dose Fucoidan P; FM, HFD with medium-dose Fucoidan P; FH, HFD with high-dose Fucoidan P; AST, aspartate transaminase; ALT, alanine aminotransferase

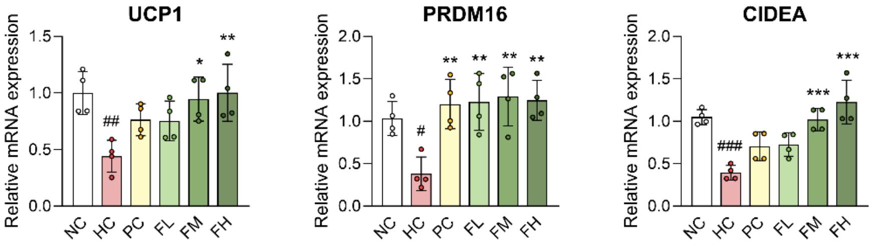

Fig. S3 Effects of Fucoidan P on fat browning in HFD-fed mice. The mice were fed an HFD for 8 weeks, and then orally administered Fucoidan P or quercetin or saline for 6 weeks. Relative expression levels of fat browning-related markers in subcutaneous fat (SuF). All results are expressed as mean ± SD. $^{\#}p < 0.05$, $^{\#\#}p < 0.01$, $^{\#\#\#}p < 0.001$ vs. NC; $^{*}p < 0.05$, $^{**}p < 0.01$, $^{***}p < 0.001$ vs. HC. NC, normal-fat diet control; HC, high-fat diet (HFD) control; PC, HFD with quercetin; FL, HFD with low-dose Fucoidan P; FM, HFD with medium-dose Fucoidan P; FH, HFD with high-dose Fucoidan P; SuF, subcutaneous fat

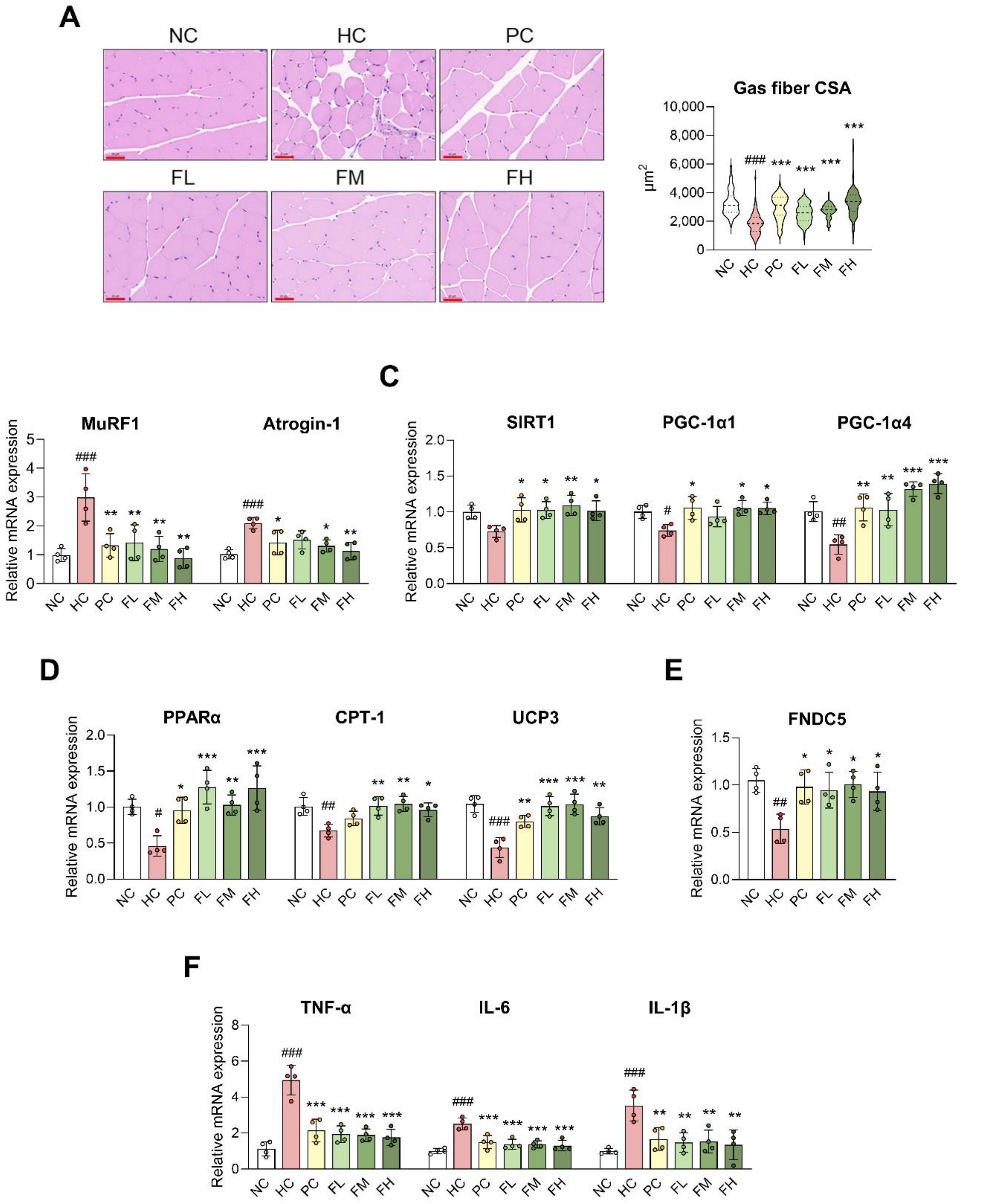

Fig. S4 Effects of Fucoidan P on sarcopenic obesity in the gastrocnemius muscle (Gas) of HFD-fed mice. The mice were fed an HFD for 8 weeks, and then orally administered Fucoidan P or quercetin or saline for 6 weeks. (A) Representative images (scale bar = 50 μm) and fiber

cross-sectional area (CSA, μm^2) from H&E staining. Relative mRNA expression levels of (B) muscle atrophy-related markers, (C) mitochondrial function-related markers, (D) fatty acid oxidation-related markers, (E) FNDC5, and (F) pro-inflammatory cytokines. All results are expressed as mean ± SD. $^{\#}p < 0.05$, $^{\#\#}p < 0.01$, $^{\#\#\#}p < 0.001$ vs. NC; $^{*}p < 0.05$, $^{**}p < 0.01$, $^{***}p < 0.001$ vs. HC. NC, normal-fat diet control; HC, high-fat diet (HFD) control; PC, HFD with quercetin; FL, HFD with low-dose Fucoidan P; FM, HFD with medium-dose Fucoidan P; FH, HFD with high-dose Fucoidan P

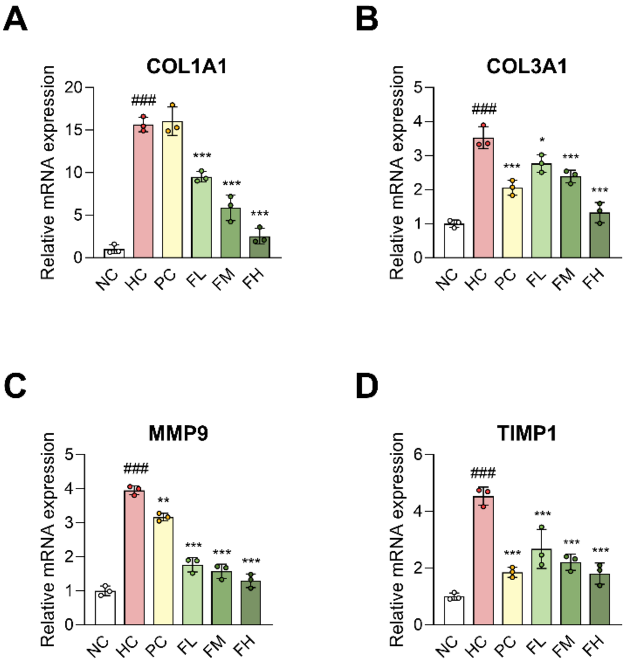

Fig. S5 Effects of Fucoidan P on extracellular matrix remodeling in HFD-fed mice. The mice were fed an HFD for 8 weeks, and then orally administered Fucoidan P or quercetin or saline for 6 weeks. The relative mRNA expression levels of (A) COL1A1, (B) COL3A1, (C) MMP9, and (D) TIMP1 in Quad. All results are expressed as mean ± SD. $^{\#\#\#}p < 0.001$ vs. NC; $^{*}p < 0.05$, $^{**}p < 0.01$, $^{***}p < 0.001$ vs. HC. NC, normal-fat diet control; HC, high-fat diet (HFD) control; PC, HFD with quercetin; FL, HFD with low-dose Fucoidan P; FM, HFD with medium-dose Fucoidan P; FH, HFD with high-dose Fucoidan P

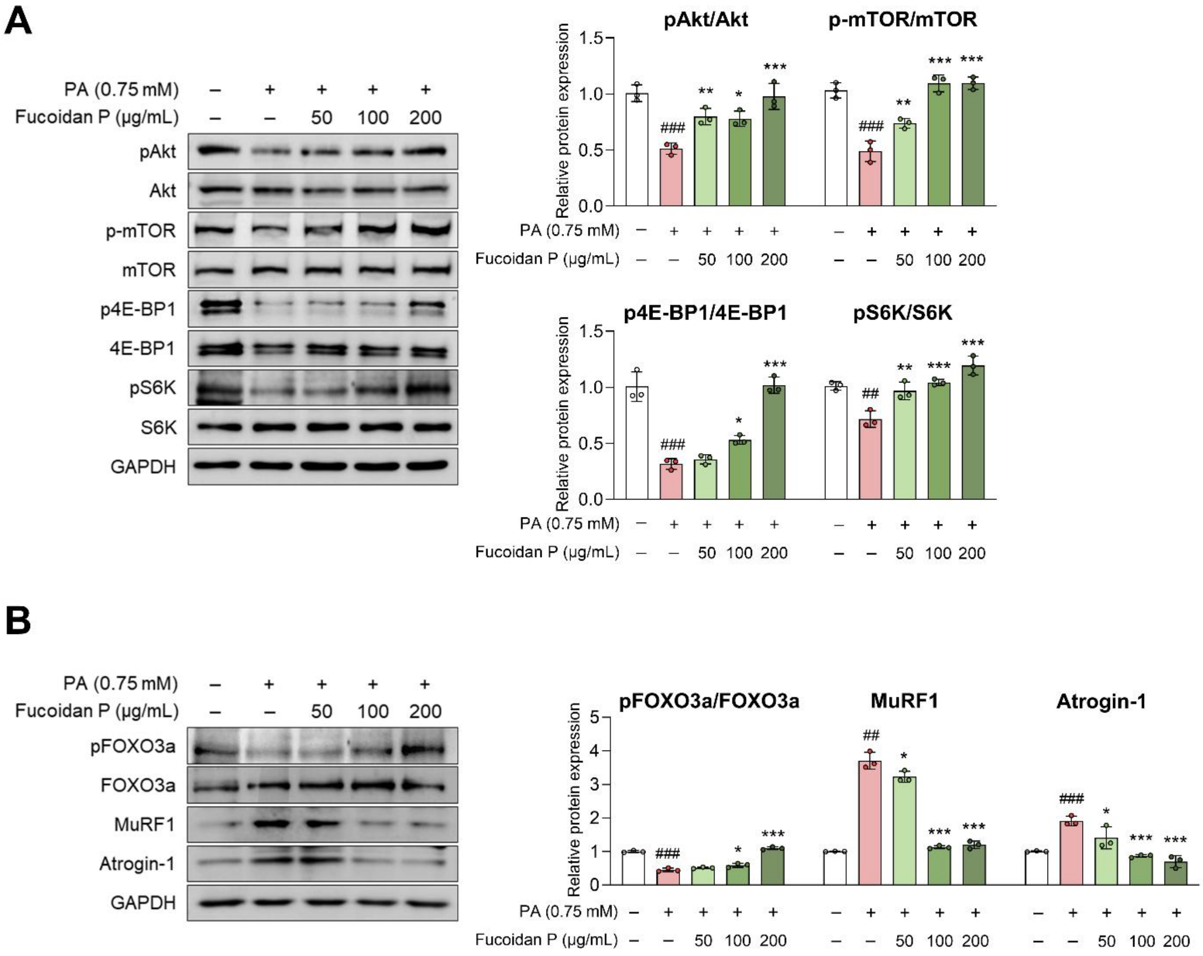

Fig. S6 Effects of Fucoidan P on muscle protein synthesis and degradation via the Akt pathway in PA-treated C2C12 myotubes. After myogenic differentiation, C2C12 myotubes were co-treated Fucoidan P and PA for 24 h. Relative protein expression levels of (A) protein synthesis-related markers and (B) protein degradation-related markers in C2C12 myotubes. All results are expressed as mean ± SD. $^{\#\#}p < 0.01$, $^{\#\#\#}p < 0.001$ vs. vehicle control; $^{*}p < 0.05$, $^{**}p < 0.01$, $^{***}p < 0.001$ vs. PA. PA, palmitate

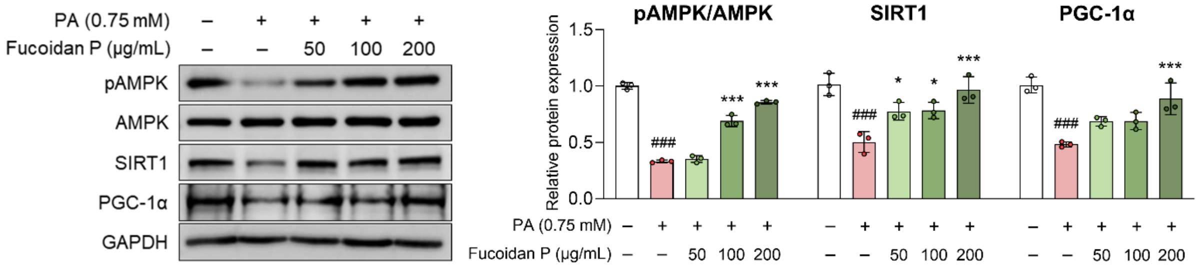

Fig. S7 Effects of Fucoidan P on energy metabolism via the AMPK/SIRT1/PGC-1α pathway in PA-treated C2C12 myotubes. After myogenic differentiation, C2C12 myotubes were co-treated Fucoidan P and PA for 24 h. Relative protein expression levels of energy metabolism-related markers in C2C12 myotubes. All results are expressed as mean ± SD. $^{###}p < 0.001$ vs. vehicle control; $^{*}p < 0.05$, $^{***}p < 0.001$ vs. PA. PA, palmitate

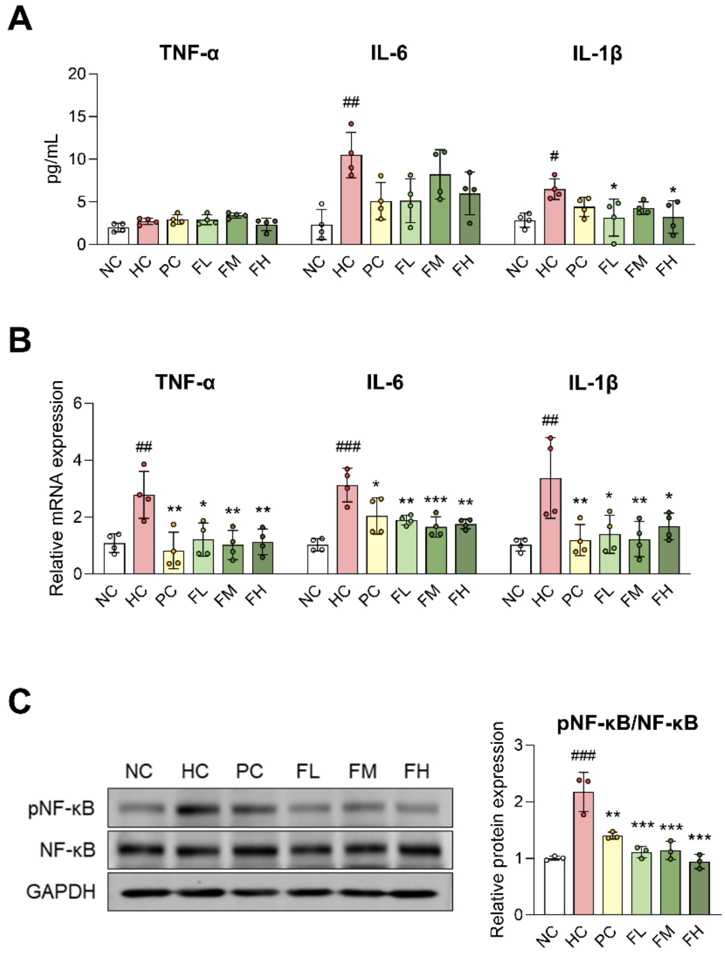

Fig. S8 Effects of Fucoidan P on inflammatory response in HFD-fed mice. The mice were fed an HFD for 8 weeks, and then orally administered Fucoidan P or quercetin or saline for 6 weeks. (A) Serum levels of pro-inflammatory cytokines. (B) Relative mRNA expression levels of pro-inflammatory cytokines in Quad. (C) Relative protein expression levels of pNF-κB/NF-κB in Quad. All results are expressed as mean ± SD. $^{\#}p < 0.05$, $^{\#\#}p < 0.01$, $^{\#\#\#}p < 0.001$ vs. NC; $^{*}p < 0.05$, $^{**}p < 0.01$, $^{***}p < 0.001$ vs. HC. NC, normal-fat diet control; HC, high-fat diet (HFD) control; PC, HFD with quercetin; FL, HFD with low-dose Fucoidan P; FM, HFD with medium-dose Fucoidan P; FH, HFD with high-dose Fucoidan P; Quad, quadriceps femoris muscle

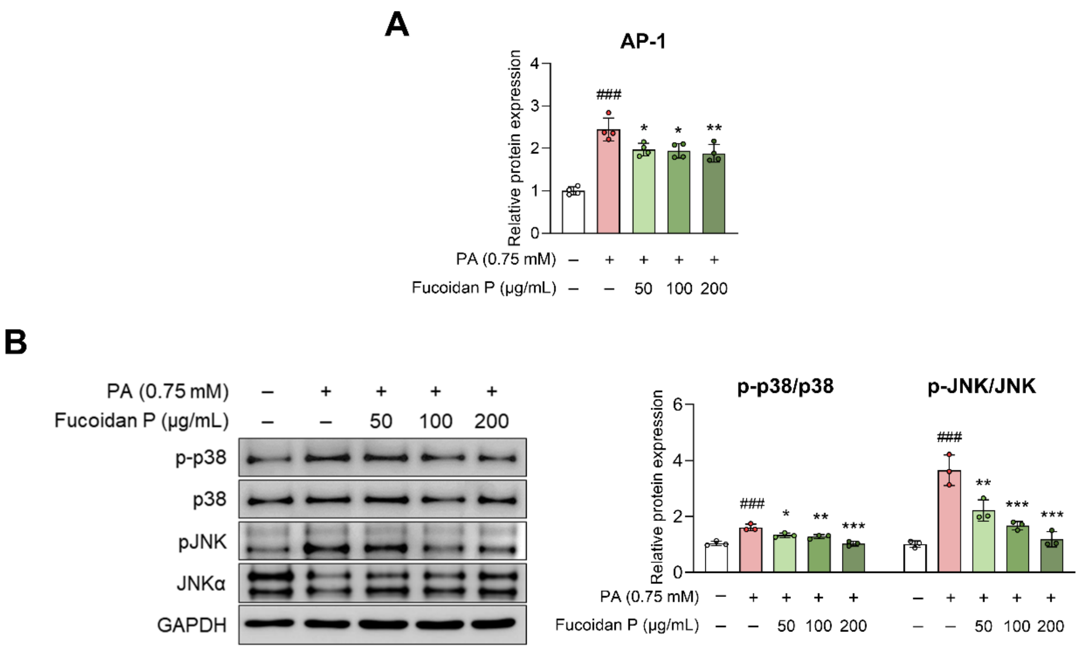

Fig. S9 Effects of Fucoidan P on MAPK inactivation in PA-treated C2C12 myotubes. After myogenic differentiation, C2C12 myotubes were co-treated Fucoidan P and PA for 24 h. (A) Relative luciferase activity of AP-1. (B) Relative protein expression levels of MAPK in C2C12 myotubes. All results are expressed as mean ± SD. $^{###}p < 0.001$ vs. vehicle control; $^{*}p < 0.05$, $^{**}p < 0.01$, $^{***}p < 0.001$ vs. PA. PA, palmitate

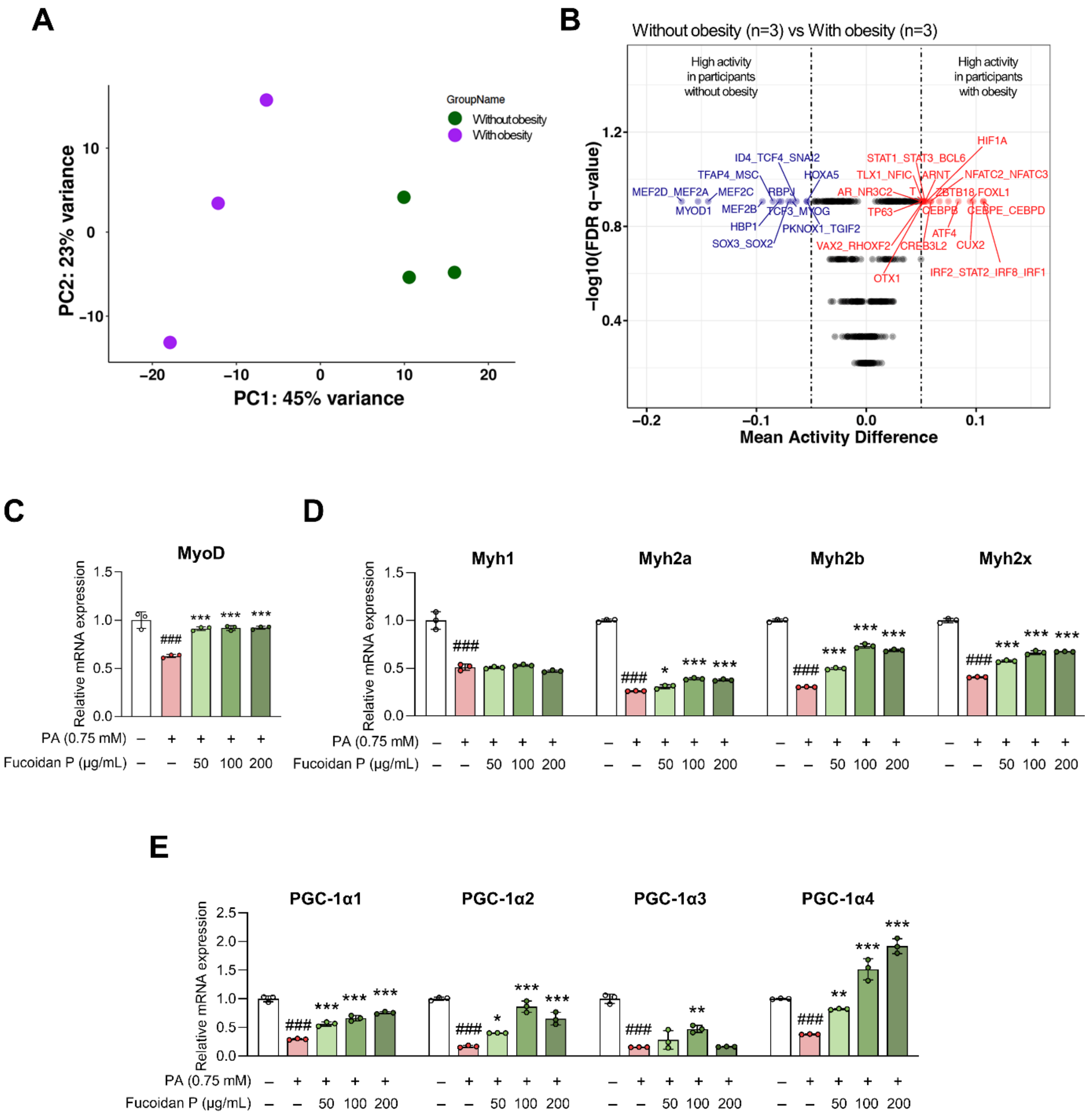

Fig. S10 Transcription factor (TF) activity between obese and non-obese groups and validation using PA-treated C2C12 myotubes. (A) Principal component analysis (PCA) of TF activities in obese and non-obese (control) groups. (B) TF activity difference between control and obese groups. The x-axis is the mean TF activity differences and the y-axis is –log10 (FDR q-value). (C-E) The target genes of MEF2D_MEF2A, the TF that showed the largest absolute mean difference. Relative expression levels of (C) MyoD, (D) myosin heavy

chain (Myh) isoforms, and (E) PGC-1α isoforms. All results are expressed as mean ± SD. $^{###}p < 0.001$ vs. vehicle control; $^{*}p < 0.05$, $^{**}p < 0.01$, $^{***}p < 0.001$ vs. PA. PA, palmitate

Supplementary Reference

S1. Balwierz PJ, Pachkov M, Arnold P, Gruber AJ, Zavolan M, Van Nimwegen E. ISMARA: automated modeling of genomic signals as a democracy of regulatory motifs. Genome research. 2014;24:869-84.
